# TNIK: A redox sensor in endothelial cell permeability

**DOI:** 10.1126/sciadv.adk6583

**Published:** 2024-12-20

**Authors:** Justin Joachim, Davide Maselli, Emmanouela Petsolari, Jurjan Aman, Pamela Swiatlowska, David Killock, Hiba Chaudhry, Ali A. Zarban, Mosharraf Sarker, Paul Fraser, Simon J. Cleary, Richard Amison, Isabelle Cuthbert, Yue Yang, Magda Meier, Franca Fraternali, Susan D. Brain, Ajay M. Shah, Aleksandar Ivetic

**Affiliations:** ^1^ School of Cardiovascular and Metabolic Medicine and Sciences, James Black Centre, BHF Centre of Research Excellence, 125 Coldharbour Lane, King’s College London, London SE5 9NU, UK.; ^2^ Randall Centre for Cell and Molecular Biology, King’s College London, London SE1 1UL, UK.; ^3^ Department of Pulmonary Medicine, Amsterdam University Medical Center, location VUMC, Amsterdam, The Netherlands.; ^4^ Myocardial Function, National Heart and Lung Institute, Imperial College London, ICTEM, Hammersmith Hospital, London, UK.; ^5^ Department of Pharmacological Sciences, Faculty of Pharmacy, Jazan University, Saudi Arabia.; ^6^ Pharmacy and Biomolecular Sciences, Faculty of Science, Liverpool John Moores University, Liverpool, UK.; ^7^ Institute of Pharmaceutical Science, King’s College London, Floor 5, Southwark Wing, Guy’s Hospital, Great Maze Pond, London SE1 9RT, UK.; ^8^ School of Cancer and Pharmaceutical Sciences, Pulmonary Pharmacology Unit, King’s College London, London, UK.; ^9^ School of Genetics and Genomic Medicine, University College London Institute of Child Health, London, UK.; ^10^ Division of Biosciences, Structural and Molecular Biology Department, University College London, Darwin (SMB) Building, Gower Street, London WC1E 6BT, UK.; ^11^ Department of Structural and Molecular Biology, Division of Biosciences and Institute of Structural and Molecular Biology, University College London, London WC1E 6BT, UK Department of Biological Sciences, Birkbeck, University of London, London WC1E 7HX, United Kingdom.

## Abstract

Dysregulation of endothelial barrier integrity can lead to vascular leak and potentially fatal oedema. TNF-α controls endothelial permeability during inflammation and requires the actin organizing Ezrin-Radixin-Moesin (ERM) proteins. We identified TRAF2 and NCK-interacting kinase (TNIK) as a kinase directly phosphorylating and activating ERM, specifically at the plasma membrane of primary human endothelial cells. TNIK mediates TNF-α–dependent cellular stiffness and paracellular gap formation in vitro and is essential in driving inflammatory oedema formation in vivo. Unlike its homologs, TNIK activity is negatively and reversibly regulated by H_2_O_2_-mediated oxidation of C202 within the kinase domain. TNIK oxidation results in intermolecular disulfide bond formation and loss of kinase activity. Pharmacologic inhibition of endogenous reactive oxygen species production in endothelial cells elevated TNIK-dependent ERM phosphorylation, endothelial cell contraction, and cell rounding. Together, we highlight an interplay between TNIK, ERM phosphorylation, and redox signalling in regulating TNF-induced endothelial cell permeability.

## Teaser

TNF activation of TNIK promotes endothelial permeability, and inhibition of TNIK reseals paracellular gap formation.

## INTRODUCTION

Endothelial cells form an essential semipermeable monolayer lining the entire vasculature, known as the endothelium, providing a regulated physical barrier between the blood or lymph and the surrounding interstitium. This endothelial barrier regulates the movement of nutrients and hormones from the blood to the organ and controls oncotic pressure through albumin transport. Substances pass from the vessel lumen to the abluminal side of the endothelium through two major routes: the transcellular and paracellular routes. Paracellular permeability occurs at endothelial cell-cell junctions, which increase significantly during inflammation and pathology (*1*–*4*). When endothelial cell-cell junctions become disassembled during inflammation, this leads to increased microvascular permeability and potentially fatal oedema formation, often called “vascular leak,” such as during acute respiratory distress syndrome (ARDS) (*5*, *6*). Endothelial hyperpermeability is also implicated in other disease states including solid tumor formation, age-related macular degeneration, and after ischemic injury (*7*).

The inflammatory cytokine tumor necrosis factor–α (TNF-α) drives endothelial permeability via activation of the Ezrin-Radixin-Moesin (ERM) proteins, which directly link the cortical actin cytoskeleton with the plasma membrane (*8*–*10*). Cytosolic ERM are held in an inactive closed confirmation, through autoinhibited interactions between the N-terminal band four-point one, ezrin, radixin, moesin (FERM) domain and C-terminal actin-binding domain of ERM (*11*–*13*). ERM activation is induced via the signalling lipid phosphatidylinositol 4,5-bisphosphate (PIP_2_) at the plasma membrane. Subsequently, the N-terminal FERM domain directly binds PIP_2_, and this precedes full ERM activation likely through conformational opening of ERM (*14*–*16*). The exposed ERM C terminus can then be phosphorylated at a conserved C-terminal threonine residue by kinases such as protein kinase C (PKC), resulting in direct binding to the cortical actin cytoskeleton and full activation of ERM (*17*–*21*). ERM therefore link the plasma membrane to the actin cortex to control several cellular functions, such as cell migration, formation of cell surface structures (e.g., microvilli), retraction of membrane blebs, and signal transduction (*9*, *22*, *23*). However, the mechanisms by which TNF-α directly induces ERM activation in the endothelium are poorly understood (*10*).

Our work reveals a redox-sensitive kinase, TRAF2 and NCK-interacting kinase (TNIK), which directly phosphorylates and activates ERM to mediate paracellular gap formation and endothelial permeability following TNF-α stimulation. We also show that paracellular gaps are resealed following treatment of TNF-activated endothelial cells with the inhibitor, KY-05009. TNIK kinase activity is reversibly inhibited by H_2_O_2_ oxidation, which occurs on C202 within the TNIK kinase domain (KD). Oxidation of C202 leads to multimerization of TNIK, which can be reversed upon thiol reduction. Pharmacologic inhibition of reactive oxygen species (ROS) production in endothelial cells was shown to augment the TNIK-ERM pathway, leading to cell retraction, paracellular gaps, and cell rounding. These observations suggest that endothelial-derived ROS may play a role in limiting the overactivation of TNIK. This work provides insights into the regulatory mechanisms that govern TNF-α–induced endothelial cell permeability.

## RESULTS

### In vitro and in cellulo p-ERM by TNIK

**Fig. 1.**
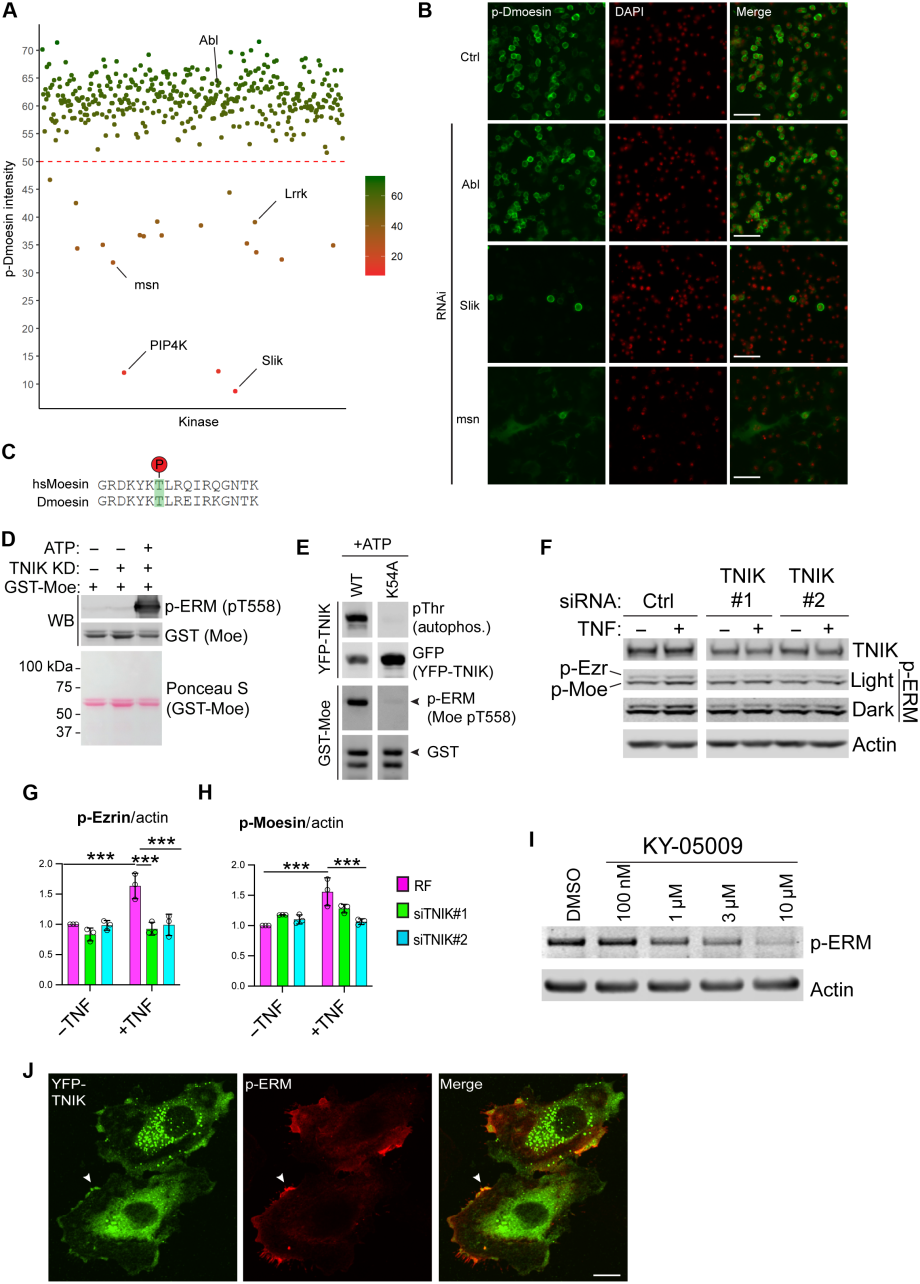
MSN and TNIK are ERM kinases in *Drosophila* and HUVEC, respectively. (**A**) Kinome-wide RNAi screen in *D. melanogaster* S2R^+^ cells. A score of p-Dmoesin staining intensity (arbitrary scale) was assigned to each kinase knockdown, with a cutoff score of ≤50 regarded as a kinase positively regulating Dmoesin phosphorylation. (**B**) Confocal images from high-content screen in (A), cells were fixed and stained with anti–p-Dmoesin (green) or 4′,6-diamidino-2-phenylindole (DAPI) (red) to stain nuclei. Scale bars, 50 μm. Ctrl, control S2R^+^ cells, not treated with dsRNA. (**C**) Alignment of *Drosophila* (D) moesin with human (hs) moesin showing the highly conserved amino acid region around Thr^558^ (human)/Thr^559^ (*Drosophila*), highlighted in green, which is detected by the anti–p-ERM antibody. (**D**) GST-TNIK KD mixed with GST-Moesin C-terminal domain and subjected to in vitro kinase assays before Western blotting. (**E**) In vitro kinase assays using full-length WT YFP-TNIK or kinase-dead K54A YFP-TNIK, followed by Western blotting as in (D). Arrowhead denotes GST-Moesin C-terminal domain. Autophos., autophosphorylation. (**F**) HUVEC transfected with control siRNA (Ctrl, RISC-free) or siRNA duplexes targeted to TNIK and stimulated with TNF-α for 15 min before Western blotting. p-Ezr, p-Ezrin. p-Moe, p-Moesin. (**G** and **H**) Quantification of (F), two-way analysis of variance (ANOVA), means ± SEM. ***P ≤ 0.001, *n* = 3 independent experiments. RF, RISC-free control siRNA. (**I**) HUVEC were treated with the indicated concentration of TNIK inhibitor KY-05009 for 2 hours before Western blotting. (**J**) HUVEC transfected with YFP-TNIK reveal that only membrane-localized TNIK is coincident with p-ERM. Scale bar, (C) 10 μm. KD, knock down; ATP, adenosine 5′-triphosphate.

ERM proteins are required for TNF-α–induced endothelial permeability in human pulmonary microvascular endothelial cells (*10*). ERM activation during TNF-α stimulation, measured by C-terminal threonine phosphorylation of ERM (p-ERM), is lost upon inhibition or small interfering RNA (siRNA) depletion of the kinases PKC, p38 mitogen-activated protein kinase, or PIP5KIα (an enzyme generating PIP_2_) (*10*). To identify kinases that phosphorylate and therefore activate ERM, we performed an unbiased kinome-wide RNA interference (RNAi) screen in *Drosophila melanogaster* cells using p-Dmoesin immunofluorescent staining as a readout (Fig. 1, A to C, and table S1). For our purposes, entire kinome screening in *Drosophila* provides certain benefits; for example, high efficiency knockdown of protein expression can be achieved using double-stranded RNA (dsRNA)–mediated RNAi and a single ERM protein is expressed in *Drosophila*, Dmoesin, which simplifies our functional readout and removes the complication of redundancy (*24*–*26*). We screened for kinases that when depleted by RNAi resulted in loss of p-Dmoesin staining using an anti–p-ERM antibody that recognises the highly conserved activating phosphorylation of pT559 (*Drosophila* Dmoesin)/pT558 (human moesin) (Fig. 1C) (*27*). RNAi of the majority of kinases, such as Abl, had no effect on p-Dmoesin staining compared with control cells (Fig. 1, A and B). RNAi of 19 kinases resulted in reduction of p-Dmoesin staining (Fig. 1A and table S1). Validating our experimental approach, protein kinases previously described to promote ERM phosphorylation were identified as hits in our screen, such as Lrrk and slik, as well as lipid kinases involved in the synthesis of PIP_2_ (or PIP_2_ precursors), such as PIP4K, Pi4KIIIα, and fwd (Fig. 1, A and B, and table S1) (*27*–*30*). Of particular interest to us, the depletion of the kinase Misshapen (msn) robustly suppressed p-Dmoesin staining (Fig. 1, A and B, and table S1). In addition, during our study, a targeted kinase screen in *Drosophila* independently identified Misshapen as a Dmoesin kinase (*31*). A human ortholog of Misshapen, TRAF2 and NCK-interacting protein kinase (TNIK), binds to the TNF receptor adaptor TRAF2, activates JNK signalling similar to TNF-α, and, controls cell shape similar to Dmoesin and Misshapen (*32*–*34*). We therefore hypothesized that in humans, TNIK phosphorylates ERM in endothelial cells during TNF-α stimulation.

To prove that TNIK directly phosphorylates ERM, we established an in vitro kinase assay using recombinant purified human TNIK KD. TNIK was able to autophosphorylate on threonine residue(s) and phosphorylate the promiscuous kinase substrate, myelin basic protein (MBP) (fig. S1A) (*35*). We next recombinantly expressed and purified the glutathione *S*-transferase (GST)–tagged C-terminal region of human moesin from *Escherichia coli* (fig. S1B). The TNIK KD was able to directly phosphorylate moesin at its C-terminal–activating T558 residue in vitro (Fig. 1D). To investigate whether full-length TNIK phosphorylates ERM, we overexpressed yellow fluorescent protein (YFP)–TNIK or the kinase-dead mutant YFP-TNIK K54A (*36*) in human embryonic kidney (HEK) 293T cells. Immunoprecipitated wild-type (WT) YFP-TNIK, but not the kinase-dead K54A mutant, was able to phosphorylate moesin in vitro (Fig. 1E). These data demonstrate that TNIK is a novel kinase directly phosphorylating and activating ERM. To test the role of endogenous TNIK in TNF-α–induced endothelial ERM activation, we used two different siRNA duplexes to deplete TNIK in primary human umbilical vein endothelial cells (HUVECs) (Fig. 1, F to H, and fig. S1C). Transfection of HUVEC with siRNA targeted against TNIK resulted in partial knockdown of TNIK expression. Stimulation of HUVEC with TNF-α led to an increase in p-ERM, which was in keeping with previous observations in human pulmonary microvascular endothelial cells (*10*). siRNA-mediated knockdown of TNIK blocked TNF-α–induced ERM phosphorylation (both ezrin and moesin) in HUVEC without affecting total ERM protein levels (Fig. 1, G and H, and fig. S1D), suggesting that TNIK is a TNF-α–inducible kinase. The adenosine 5′-triphosphate (ATP)–competitive TNIK inhibitors KY-05009, NCB-0846, and PF-794 were able to more robustly inhibit TNIK function and ERM phosphorylation in the endothelium (Fig. 1I and fig. S1, E to G) (*37*–*39*).

YFP-TNIK expressed in HUVEC localized to the plasma membrane, plasma membrane blebs, punctate cytoplasmic structures, and diffuse cytoplasmic structures (Fig. 1J and fig. S1, H and I). Phosphorylated and active ERM colocalized with YFP-TNIK at the plasma membrane or plasma membrane blebs (Fig. 1J and fig. S1, H and I). Moreover, HUVEC expressing YFP-TNIK had markedly higher amounts of plasma membrane p-ERM than neighboring cells expressing low or negligible amounts of YFP-TNIK (fig. S1I). In contrast to WT YFP-TNIK, kinase-dead K54A YFP-TNIK exhibited a mostly punctate cytoplasmic localization in HUVEC with little to no colocalization with p-ERM (fig. S1J). Endothelial cells expressing YFP-TNIK K54A had similar levels of p-ERM compared with neighboring cells expressing low or negligible amounts of YFP-TNIK K54A (fig. S1J). These data suggest that TNIK preferentially phosphorylates ERM at the plasma membrane of endothelial cells. Moreover, we show that TNIK is a TNF-α–responsive ERM kinase in endothelial cells.

### TNIK regulates endothelial permeability and paracellular gaps in vitro and inflammatory oedema formation in vivo

**Fig. 2.**
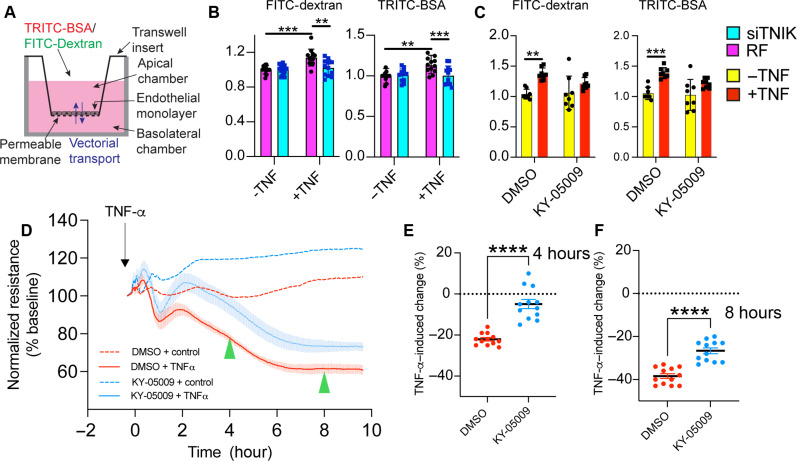
TNIK positively regulates TNF-α–induced permeability and KY-05009 reverses permeability after TNF-α stimulation. (**A**) Schematic illustrating transwell in vitro endothelial permeability assay (see also Materials and Methods). (**B**) HUVEC monolayers were treated with RISC-free (RF) control siRNA or TNIK siRNA before transwell permeability assays. Monolayers were stimulated for 4 hours with TNF-α, and fluorescent tracers (67 kDa tetramethyl rhodamine isothiocyanate (TRITC)–bovine serum albumin (BSA) and 4 kDa fluorescein isothiocyanate (FITC)–dextran) were subsequently added for a further hour before detection of fluorescence emerging in the lower chamber (see Materials and Methods). Statistical analysis using two-way ANOVA, means ± SEM, ***P ≤ 0.001, four independent experiments. (**C**) HUVEC monolayers were stimulated with TNF-α for 4 hours, followed by the addition of fluorescein isothiocyanate (FITC)–dextran or tetramethyl rhodamine isothiocyanate (TRITC)–bovine serum albumin (BSA), alongside dimethyl sulfoxide (DMSO) or 10 μM KY-05009 for a further 1 hour to monitor resealing or reversal of permeability (see Materials and Methods). Statistical analysis using two-way ANOVA, means ± SEM, **P ≤ 0.01 and ***P ≤ 0.001, four independent experiments. (**D**) Evaluation of the effect of KY-05009 on endothelial barrier integrity using endothelial cell-substrate impedance sensing. Cells were pretreated with 10 μM KY-05009 (2 hours) followed by addition of TNF-α (10 ng/ml, arrow). Resistance values were normalized to baseline (see fig. S2A for non-normalized graph). Green arrowheads indicate where resistance was decreasing mid-point at 4 hours and where resistance was plateauing at 8 hours. (**E** and **F**) Graphs of the normalized resistance trace represent TNF-α induced drop in endothelial resistance as compared to baseline at *t* = 4 hours and *t* = 8 hours. Mean and 95% confidence interval of 12 biological repeats from *n* = 4 independent experiments. Unpaired Student’s *t* test, **P ≤ 0.0013 and ****P ≤ 0.0001.

**Fig. 3.**
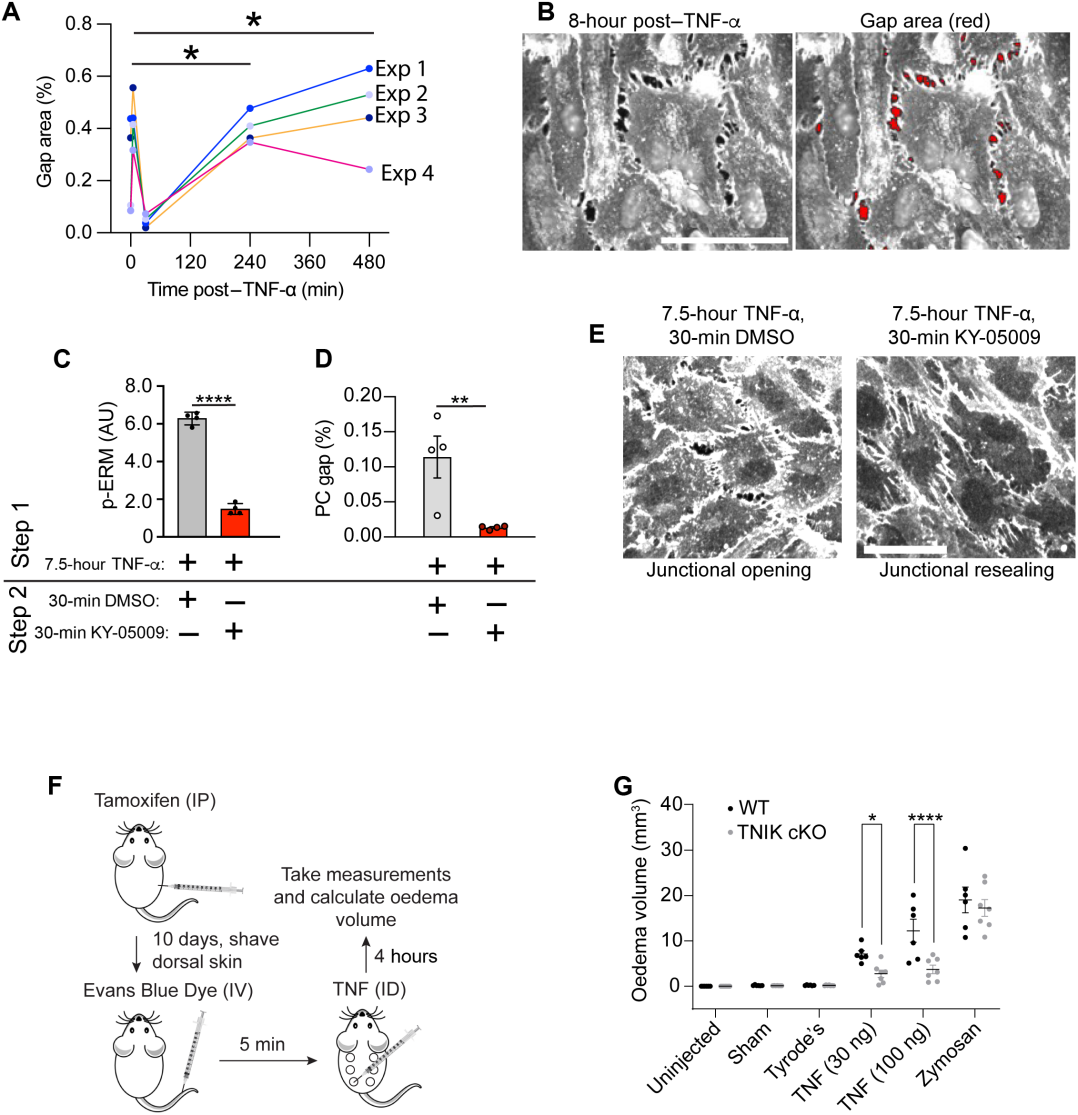
TNIK positively regulates TNF-α–induced paracellular gap formation in vitro, and TNF-α–induced oedema formation in vivo. (**A**) Time course of HUVEC gap formation in response to TNF-α stimulation. Four fields of view (FoV) were acquired per experiment (see fig. S2B for representative FoV). Data represent *n* = 4 and are expressed as a percentage of gaps per FoV. Exp, experiment. Data analyzed using a repeated measures one-way ANOVA with Dunnett’s test for differences relative to unstimulated cells: *P ≤ 0.05. (**B**) Two images showing approach for quantifying endothelial gaps (see Materials and Methods for more details). Scale bar, 50 μm. (**C**) Quantification of *n* = 4 p-ERM Western blots of HUVEC monolayers treated with TNF-α for 7.5 hours, followed by 30 min with KY-05009 or DMSO carrier. Unpaired Student’s *t* test, ****P ≤ 0.0001. (**D**) Paracellular (PC) gaps were quantified as indicated in left image of (B). Paracellular gaps are resealed after addition of KY-05009. (**E**) Representative images of data acquired in (D), showing resealing of paracellular gaps (see fig. S2C for larger FoV). Scale bar, 50 μm. (**F**) Schematic of in vivo skin plasma extravasation assay (see also Materials and Methods). IP, intraperitoneal injection. IV, intravenous injection. ID, intradermal injection. (**G**) *Tnik* endothelial-specific conditional knockout mice were subjected to skin permeability assays. *n* = 6 to 7 mice per group. Statistical analysis using two-way ANOVA, means ± SEM, *P ≤ 0.05, **P ≤ 0.01, and ****P ≤ 0.0001.

We next conducted a series of in vitro and in vivo experiments to determine whether TNIK regulates endothelial cell permeability and paracellular gap formation. We first used transwell endothelial permeability assays to investigate whether TNIK regulation of paracellular gaps and cell-cell contacts affects permeability of the endothelium to macromolecules. Treatment of the endothelium with TNIK siRNA significantly inhibited TNF-α-induced endothelial permeability to albumin (67 kDa) and dextran (4 kDa) (Fig. 2, A and B). Moreover, treatment of the endothelium with 10 μM KY-05009, after 4 hours of stimulation with TNF-α, suppressed permeability to albumin or dextran, suggesting resealing or reversal of permeability (Fig. 2, A to C). These results suggest that TNIK promotes TNF-α–induced endothelial paracellular permeability through the retraction of apposing endothelial cell membranes and that KY-05009 can promote paracellular gap resealing. Electric cell-substrate impedance sensing (ECIS; see Methods) allowed for continuous monitoring of TNF-α–induced barrier disruption over a 10-hour period. TNF-α–induced permeability was significantly suppressed by 10 μM KY-05009 between the 2- and 10-hour time points (Fig. 2, D to F). We used confocal microscopy to determine whether paracellular gaps were forming at time points where permeability was witnessed by ECIS. Very few paracellular gaps were detected at 30 min, which increased significantly at the 4- and 8-hour time points (Fig. 3, A and B, and fig. S2, A and B). Treatment of HUVEC with 7.5-hour TNF-α, followed by 30-min exposure to KY-05009, significantly reduced ERM phosphorylation and resealing of paracellular gaps (Fig. 3, C to E, and fig. S2, B to D). We also noted that paracellular gaps were visible at the 5-min time point (Fig. 3A and fig. S2A); however, permeability was not detectable by ECIS, possibly due to rapid and unstable gap formation.

We also addressed the in vivo role of TNIK in regulating TNF-α–induced permeability by subjecting mice to a Miles Assay [see Methods, (*40*, *41*), and Fig. 3F]. Significant oedema formation occurred in response to TNF-α and to zymosan administration, a glucan originating from yeast cell walls and an activator of multiple inflammatory signaling pathways stimulating oedema formation (*42*). The TNIK inhibitor NCB-0846, which has previously been administered in mice at similar doses (*38*), robustly inhibited oedema formation induced after 4-hour TNF-α administration but not zymosan-induced oedema formation (fig. S3, A to C). Furthermore, endothelial-specific conditional *Tnik* knockout mice exhibited significant reduction in TNF-α–induced oedema formation (see Methods; Fig. 3, F and G; and fig. S3, D to F). Neutrophils are known to play an instrumental role in regulating TNF-α–induced microvascular permeability (*43*). As anticipated, endothelial-specific knockout of *Tnik* did not impair neutrophil recruitment to sites of oedema formation as measured by myeloperoxidase (MPO) activity assay of skin biopsies (fig. S3G). These data suggest that knockout of *Tnik* in endothelial cells is sufficient to suppress TNF-α–induced oedema formation, without affecting neutrophil trafficking and transendothelial migration. Together, these data strongly suggest that TNIK is essential for driving TNF-α–induced endothelial paracellular gaps and permeability that leads to in vivo oedema formation.

### Temporal assessment of TNF-α–induced p-ERM in relation to endothelial cell stiffness, paracellular gaps and permeability

**Fig. 4.**
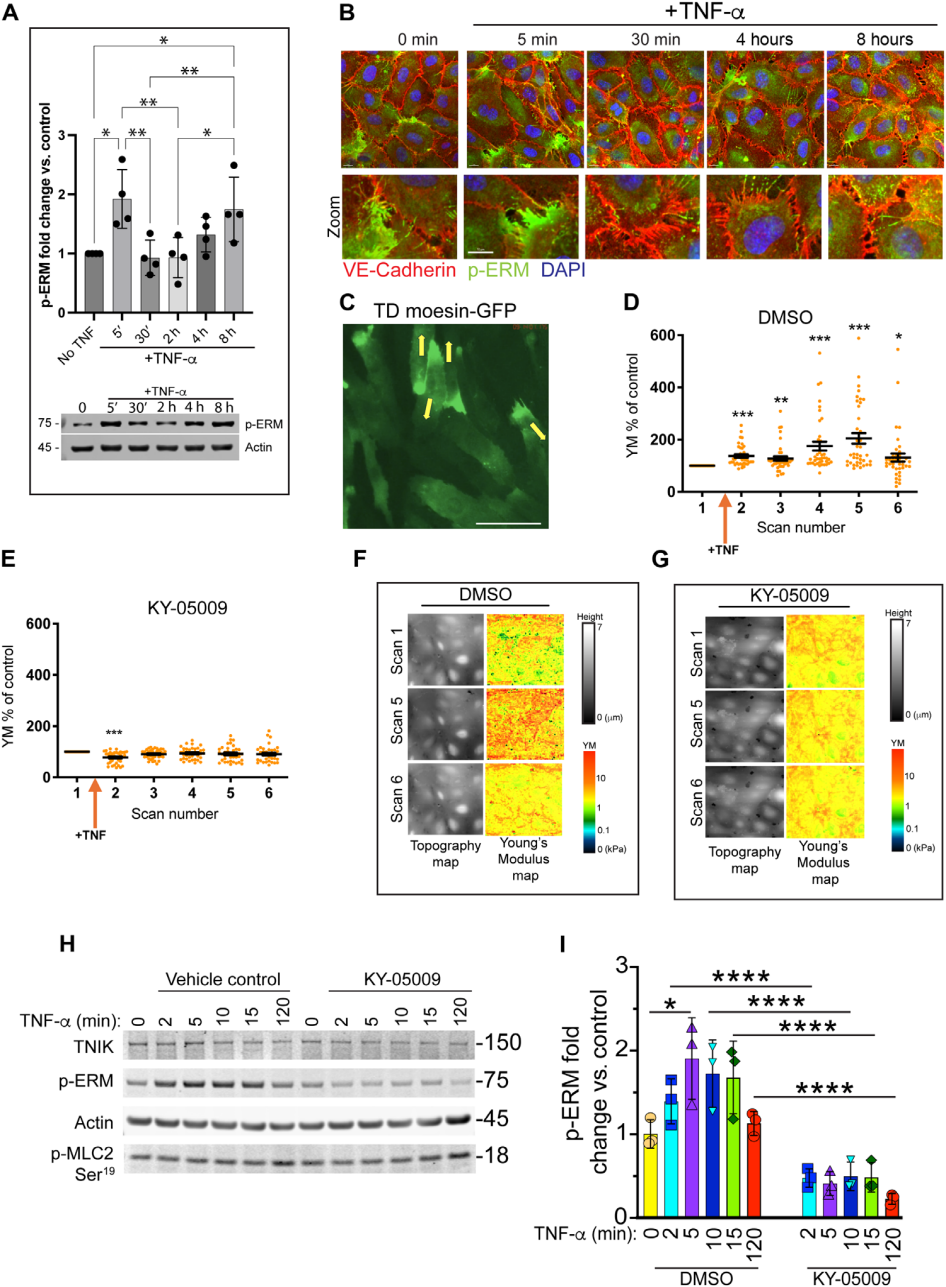
TNF-α–induced activation of HUVEC leads to bimodal ERM phosphorylation, contributing to cellular stiffness at 5 min and stable paracellular gaps at 4 to 8 hours. (**A**) HUVECs were stimulated with TNF-α (10 ng/ml) at indicated time points, and whole-cell lysates were harvested for Western blotting for p-ERM. Data are from *n* = 4 independent experiments. Western blot below bar graph is representative of the data. One-way ANOVA with Tukey’s post hoc test *P ≤ 0.05 and **P ≤ 0.01. (**B**) Confocal images of HUVEC before and after TNF-α stimulation. Fluorescence signals corresponding to VE-cadherin (red), p-ERM (green), and DNA (DAPI, blue). Scale bar, 10 μm. (**C**) Still taken from movie S1: HUVEC transduced with lentivirus to express T558D (TD) moesin-GFP, a phospho-mimic mutant of moesin (*98*). Yellow arrows indicate the direction of migration. Scale bar, 66 μm. (**D** and **E**) Quantification of Young’s Modulus (YM) of control (i.e., before TNF-α treatment). −/+ TNF-α treatment in the presence of DMSO (carrier) or KY-05009. Mean whole-cell YM values were plotted over time, approximately 21 min (i.e., 3.5 min per scan number). The first scan was acquired before adding TNF-α and DMSO/KY-05009. Values are represented as a change from control. *N*/*n* = 3/38 to 40. Data are presented as means ± SEM, Kruskal-Wallis test, *P < 0.05, **P < 0.01, and ***P < 0.001. (**F** and **G**) Representative 100 μm by 100 μm SICM images of topography and YM maps using the loop mode. (**H**) Western blot of p-ERM, performed similarly to (A), but separate series of experiments altogether, showing acute rises in p-ERM. p-MLC2 Ser^19^ = phospho myosin light chain kinase at position serine-19. Actin loading control and total TNIK protein are both represented. (**I**) Quantification of (H), *n* = 3. Two-way ANOVA with Tukey’s post hoc test. *P ≤ 0.05 and ****P ≤ 0.0001. h, hours.

To better understand the relationship between p-ERM with endothelial cell permeability and paracellular gap formation, we performed Western blotting to monitor total p-ERM levels in TNF-α–activated HUVEC across an 8-hour period (Fig. 4A). A bimodal pattern of p-ERM was observed, where levels significantly increased at 5-min and 8-hour post–TNF-α stimulation. Confocal microscopic imaging showed that the untreated and 5-min TNF-α–treated HUVEC displayed a polarized distribution of p-ERM, which was less apparent at the 4- or 8-hour time points (Fig. 4B). Transducing HUVEC with a constitutively pseudo-phosphorylated (T558D) mutant of moesin–green fluorescent protein (GFP) led to polarized redistribution to the rear of migrating HUVEC (Fig. 4C and movie S1), suggesting that HUVEC containing a polarized distribution of p-ERM are cells likely to be captured in migration. To our knowledge, acute increases in permeability (i.e., 5 min) following TNF-α stimulation has not been reported. HUVEC are, however, reported to increase in cellular tension as early as 4-min post–TNF-α stimulation (*44*). In addition, increases in p-ERM are causal to cell stiffness and tension in leukocytes and epithelial cells (*45*–*47*), and we therefore asked if the acute rise in p-ERM, 5-min post-TNF, relates to increases in cellular stiffness. Mechano scanning ion conductance microscopy (mechanoSICM) revealed that TNF-α stimulation of HUVEC led to an acute rise in cellular stiffness, within 5 to 10 min, which was TNIK dependent (Fig. 4, D to G). Western blotting also showed that acute rises in p-ERM levels coincided temporally with cellular stiffness, which was inhibited by KY-05009 (Fig. 4, H and I). The stiffness of HUVEC over acute periods of TNF-α stimulation was shown, biochemically, to be independent of myosin light chain phosphorylation (p-MLC2), which appeared to increase at later time points (i.e., at 2 hours; Fig. 4H). We therefore excluded the possibility that acute increase in p-ERM contributes to permeability but likely influences cell stiffness and may lead to transient and unstable paracellular gap formation (see Discussion). Together, our data suggest that TNIK phosphorylates ERM 5- to 10-min post–TNF-α stimulation to regulate endothelial cell stiffness and at 4- to 8-hour post–TNF-α stimulation to stabilise paracellular gaps and promote permeability.

### Membrane-associated endothelial TNIK undergoes reversible oxidation through C202

**Fig. 5.**
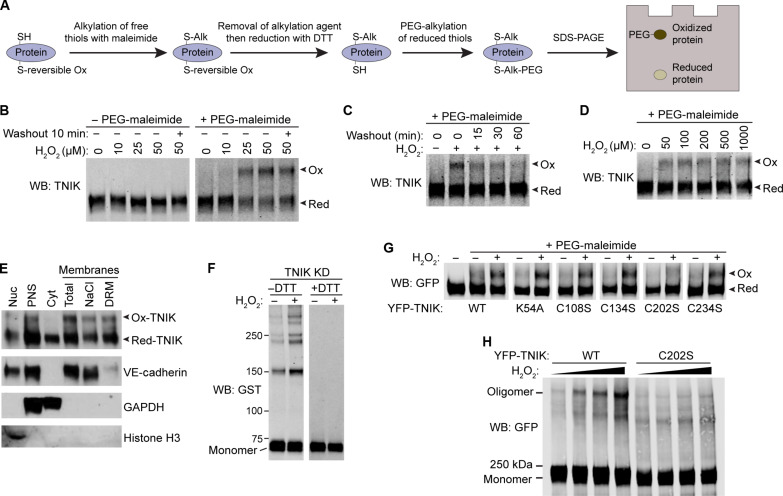
Membrane-localized TNIK undergoes reversible oxidation through C202. (**A**) Schematic of PEG-switch assay (*51*). (**B**) HUVEC monolayers were treated with hydrogen peroxide (H_2_O_2_) for 15 min and washed into EGM-2 medium without H_2_O_2_ for 10 min before PEG-switch assays and Western blotting. Bands corresponding to oxidized (Ox) and reduced (Red) forms of endogenous TNIK are indicated. (**C**) HUVEC monolayers were treated with 50 μM H_2_O_2_ for 15 min and washed into EGM-2 medium without H_2_O_2_ for the indicated time before PEG-switch assays and Western blotting. Bands corresponding to oxidized and reduced forms of endogenous TNIKs are indicated with arrowheads. (**D**) HUVEC monolayers were treated with H_2_O_2_ for 15 min before PEG-switch assays and Western blotting. Bands corresponding to oxidized and reduced forms of endogenous TNIK are indicated. (**E**) HUVEC monolayers were treated with 200 μM H_2_O_2_ for 15 min, before subcellular fractionation, PEG-switch assays and Western blotting. Nuc, nuclear pellet. PNS, post-nuclear supernatant. Cyt, cytosol. NaCl, 500 mM NaCl washed membranes. DRM, 1% Triton X-100 4°C detergent resistant membranes. Bands corresponding to oxidized and reduced forms of endogenous TNIK are indicated with arrowheads. (**F**) Recombinant GST-tagged TNIK KD was oxidized in vitro with 50 μM H_2_O_2_ at 30°C for 15 min followed by reducing (+DTT) or nonreducing (−DTT) SDS-PAGE and Western blotting. (**G**) YFP-TNIK or YFP-TNIK mutants were expressed in HEK293T cells by plasmid transfection and immunoprecipitated. Immunoprecipitates were oxidized with 200 μM H_2_O_2_ at 30°C for 15 min followed by PEG-switch assays and Western blotting. Oxidised/reduced forms of YFP-TNIK are indicated. (**H**) YFP-TNIK or YFP-TNIK C202S were expressed in HEK293T cells and immunoprecipitated. Immunoprecipitates were oxidized with increasing amounts of H_2_O_2_: 0, 0.2, 0.5, and 1 mM at 30°C for 15 min followed by nonreducing SDS-PAGE and Western blotting. WB, Western blot;

That paracellular gap formation and p-ERM levels are both low at 30-min post–TNF-α stimulation (Figs. 3A and 4A) suggests that TNIK activity could be reduced/inactivated from the 5-min time point and therefore regulated posttranslationally. The kinase activities of the TNIK homologs, MINK1 and MAP4K4, are activated by H_2_O_2_ treatment of cell cultures (*48*–*50*). We therefore hypothesized that TNIK may undergo similar modes of redox regulation. To investigate whether endogenous endothelial TNIK undergoes reversible cysteine oxidation, we performed polyethylene glycol (PEG)–switch assays, as previously described (Fig. 5A) (*51*). This technique does not detect terminally and irreversibly oxidized cysteines, such as modification of thiol side chains to sulfonic acid groups, which are less relevant for signaling, as these signals often lead to protein instability and degradation (*52*). We detected reversibly oxidized endogenous TNIK (ox-TNIK) after treating HUVEC with biologically relevant amounts of H_2_O_2_ (Fig. 5, B to D) (*52*). Oxidation of TNIK, followed by removal of H_2_O_2_, resulted in the gradual loss of ox-TNIK over the course of 1 hour, suggesting that a mechanism for TNIK regulation exists in the endothelium and that the oxidation of TNIK is reversible. We noticed that much of the endothelial TNIK population remains in a reduced state during H_2_O_2_-driven oxidation (Fig. 5, B to D). Treating the endothelium with biologically high amounts (*52*) of H_2_O_2_ (e.g., 1 mM) did not drive a corresponding increase in the proportion of detectable ox-TNIK (Fig. 5D). We reasoned that the redox-sensitive population of endothelial TNIK may reside in a specific subcellular compartment, whereas TNIK in other subcellular locations may not be redox sensitive. Subcellular fractionation revealed that ox-TNIK is enriched on endothelial membranes, whereas low or nondetectable amounts of ox-TNIK is localized in the cell cytosol and nucleus (Fig. 5E). Moreover, H_2_O_2_ induced the relocalization of the YFP-TNIK to intracellular membrane foci, which were reminiscent of the punctate localization of kinase inactive YFP-TNIK K54A (movie S2 and fig. S1J).

Reversible oxidation of cysteine thiols occurs through a labile sulfenic acid intermediate and can result in a variety of end products, such as a sulfenyl amide, glutathionylation, and intra- or intermolecular disulfide bond formation (*53*). In vitro oxidation of TNIK KD with H_2_O_2_ resulted in an increase in dimeric and oligomeric TNIK, which were detectable by nonreducing SDS–polyacrylamide gel electrophoresis (SDS-PAGE) but were not detectable by reducing SDS-PAGE after boiling with dithiothreitol (DTT), which reduces disulfide bonds (Fig. 5F). We also observed TNIK dimers and oligomers in the absence of H_2_O_2_, which we believe is due to air oxidation of TNIK (Fig. 5F). To identify which of the five cysteines within the TNIK KD can undergo reversible oxidation, we analyzed the TNIK crystal structure [Protein Data Bank (PDB): 5CWZ] with the Cy-preds Cys Oxidation Prediction Algorithm (COPA) algorithm (*54*, *55*). Cy-preds predicted TNIK C202 and C234 as potentially reactive cysteines, with C202 having a predicted low pKa of 5.61, amenable for reactivity. The five cysteine residues within the TNIK KD were serially mutated to serines by site-directed mutagenesis: C108S, C134S, C202S, C234S, and C269S (Fig. 5G). Only mutation of C202S resulted in the loss of detectable ox-TNIK in PEG-switch assays (Fig. 5G); in contrast, WT and kinase-dead K54A YFP-TNIK were both oxidizable, suggesting that oxidation of TNIK is independent of kinase activity (Fig. 5G). Using nonreducing SDS-PAGE, we found that upon oxidation, immunoprecipitated WT YFP-TNIK, but not YFP-TNIK C202S, formed H_2_O_2_ dose-dependent oligomers (Fig. 5H and fig. S4A). Together, these results suggest that TNIK on endothelial membranes is sensitive to H_2_O_2_-driven oxidation on C202 within the TNIK KD. Oxidation of TNIK likely results in intermolecular disulfide bond formation, directly involving C202. In addition, the formation of disulfide-linked oligomers larger than dimers suggests that unidentified cysteine residues other than C202 may be involved in TNIK homo/hetero-oligomerisation.

**Fig. 6.**
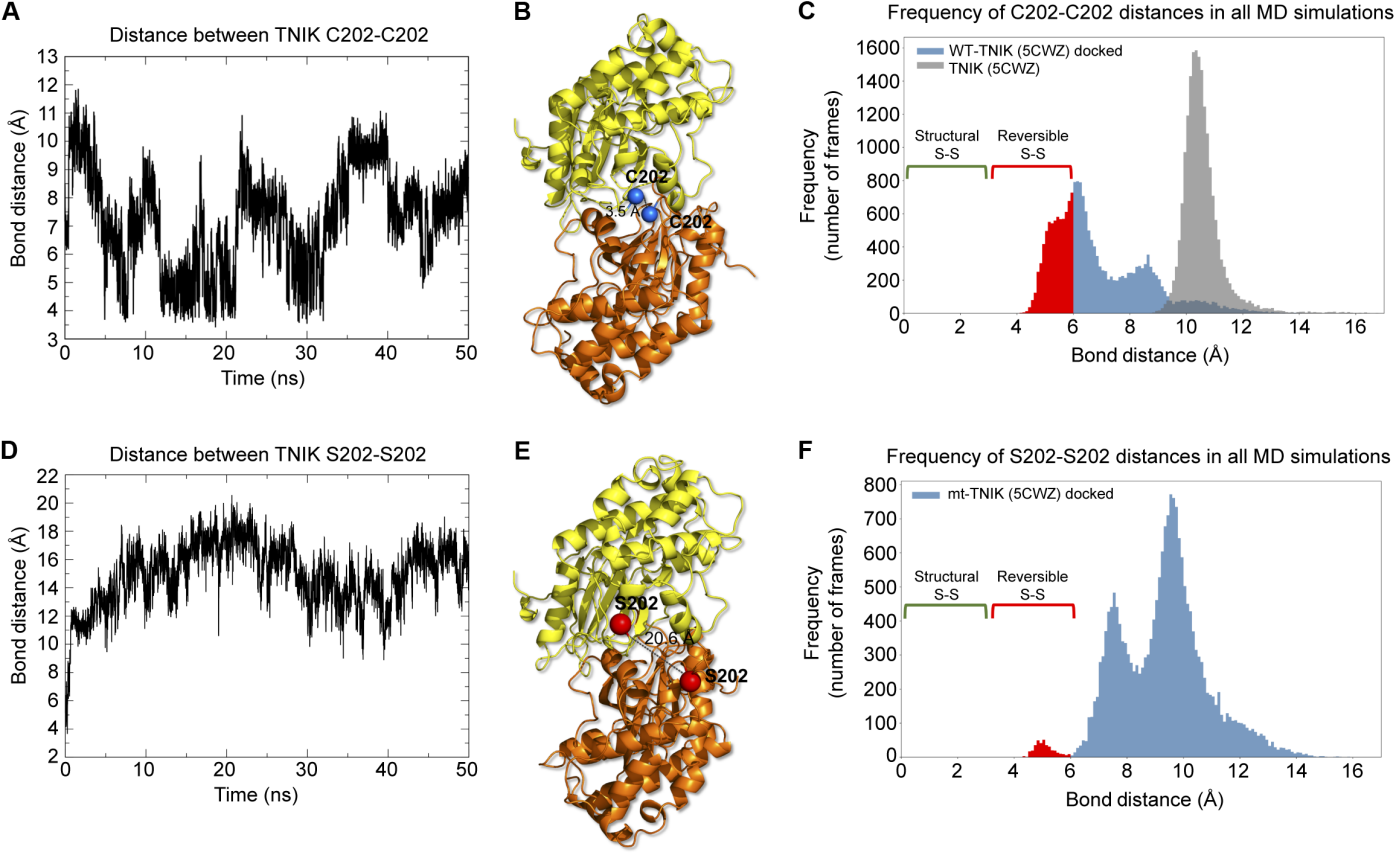
MD simulations predict apposed TNIK C202 residues in the TNIK dimer are able to form reversible disulfide bonds. (**A**) Distance between apposed C202 residue sulphur atoms in WT-TNIK docked dimer during 50-ns MD simulation. See also movie S4. Relates to fig. S4C, replica 1. (**B**) 3D structure of the minimum distance between C202-C202, retrieved from MD simulation in (A). (**C**) Frequency (number of frames) from MD simulations of the TNIK dimer where apposed C202-C202 distances are within the reversible disulfide bond range (>3.0 Å and ≤6.2 Å) (*54*) (red). The distances between apposed C202 residues in the WT-TNIK crystal structure dimer (PDB: 5CWZ) were calculated from an extended simulation trajectory of 124 ns (gray). The C202-C202 distances for the wt-TNIK docked model (blue) were calculated using a concatenated trajectory containing all four simulations from fig. S4C and 20,000 frames in total. (**D**) Distance between apposed in silico mutated S202 residues (side-chain hydroxyl hydrogen atoms) in mt-TNIK–docked dimer during 50-ns MD simulation. See also movie S5. Relates to fig. S4F, replica 4. (**E**) 3D structure of the maximum distance between S202-S202, retrieved from MD simulation in (D). (**F**) Frequency (number of frames) from MD simulations of the mt-TNIK–docked dimer where apposed in silico mutated S202-S202 distances are within the reversible disulfide bond range (>3.0 Å and ≤6.2 Å) (*54*) (red). The S202-S202 distances for the mt-TNIK–docked model (blue) were calculated using a concatenated trajectory containing all four simulations from fig. S4F and 20,000 frames in total.

To understand how TNIK C202 is involved in reversible disulfide bond formation, we examined the crystal structure of the TNIK KD (PDB: 5CWZ) (*38*). We noticed that the TNIK KD forms crystallized homodimers with adjacent C202 residue side chains, located on loop regions, 11.8 Å apart from one another (fig. S4B and movie S3). TNIK homodimers are predicted to biologically exist in solution [PDBePISA algorithm (*56*)] and have been observed experimentally (*57*). We performed molecular dynamics (MD) simulations to investigate if, in a motile system, adjacent TNIK C202-C202 residues may come close enough to allow disulfide bond formation (Fig. 6, A to C; and fig. S4, C to E; and movies S3 and S4). A disulfide bridging threshold of 6.2 Å has been reported as the maximum distance allowed for S─S bond formation (*54*). A distance of 3.0 to 6.2 Å predicts formation of reversible disulfides, which are often involved in protein regulation, and a distance <3.0 Å predicts permanent structural disulfides (*54*). We conducted a 124-ns simulation run in the TNIK crystal structure (PDB: 5CWZ) to investigate whether adjacent C202 residues could come in a close enough proximity to potentially form a disulfide bond (Fig. 6C). In this simulation C202-C202 distances reached a minimum distance of around 9 Å (Fig. 6C). For this reason, we docked the crystal structure to identify whether a small structural rearrangement could enable the reduction of the C202-C202 distance. In replicated 50-ns MD simulations of the docked TNIK structure, a C202-C202 distance of <6.0 Å was observed at several time points during the simulation trajectories, and a minimum distance of 3.54 Å was observed, consistent with distances required for reversible disulfide bond formation between TNIK C202-C202 (Fig. 6, A to C; fig. S4C; and movies S3 and S4). We concatenated all four replica simulations generated from the TNIK-docked structure and computed the frequency of C202-C202 distances (Fig. 6C and fig. S4C). This analysis shows that 35% (7032 frames of 20,000) of C202-C202 distances are within the range consistent with reversible disulfide bond formation (*54*). Further analyses of the MD simulations suggest that the simulated structures represent stable TNIK dimers that do not undergo significant global conformational changes. First, we computed the distance of the centre of mass (COM) from one TNIK KD monomer to the COM of the other monomer to map the conformation transition of the dimer during MD simulation (fig. S4D). The COM distances between the two monomers do not show a significant deviation (replicas 1 to 3), and their COM distance profiles remained stable (fig. S4D). Second, low and stable root mean square deviations (RMSDs) from the initial starting structure during each simulation were observed (fig. S4E).

Mutation of TNIK C202 to S202 prevents reversible cysteine oxidation and oligomerization and therefore likely disrupts disulfide bond formation (Fig. 5, G and H, and fig. S4A). We introduced C202S mutations in silico into both TNIK KD monomers of the docked TNIK model and conducted 50-ns MD simulations (Fig. 6, D to F; fig. S4F; and movie S5). As expected, the S202-S202 residue pair is consistently further away in three-dimensional (3D) space compared with simulations of C202-C202 and beyond the range expected for disulfide bond formation (*54*), reaching a maximum distance of up to 20.6 Å (Fig. 6, D to F; fig. S4F; and movie S5). Concatenation of the four replica simulations generated from the mt-TNIK–docked structure shows that in contrast to WT TNIK, only 1.8% (366 frames of 20,000) of S202-S202 distances are within the range consistent with reversible disulfide bond formation (compare Fig. 6, C and F). Together, our experimental and in silico data provide strong evidence that oxidation of TNIK C202 results in reversible disulfide bond formation.

To understand how TNIK C202 may regulate kinase activity, we compared the published structures of the inactive (PDB: 5CWZ) and active (PDB: 5AX9) TNIK KDs (fig. S4, G and H) (*38*). TNIK adopts a canonical KD structure, which includes an N-terminal lobe and a C-terminal lobe connected by a flexible hinge region (movie S6) (*58*). In the active TNIK structure, the αC helix within the N-lobe rotates inward, toward the ATP-binding pocket (forming the αC helix “in” conformation) and facilitates the formation of a conserved salt-bridge present among kinases, between a lysine in the β3 strand (K54 in TNIK) and a glutamate in the αC helix (E69 in TNIK) (fig. S4H and movie S6) (*38*, *58*). The formation of this salt-bridge is key for enzyme activity as it facilitates substrate access and ATP binding. The constitutively inactive K54A kinase-dead TNIK mutant can undergo reversible cysteine oxidation (Fig. 5G), and therefore, we hypothesized that oxidation and disulfide formation may occur on, and perhaps stabilize, the inactive conformation of TNIK. We noticed that only the inactive TNIK crystal structure (PDB: 5CWZ) contains juxtaposed C202-C202 residues, and we thus hypothesized that cysteine oxidation of TNIK may negatively regulate kinase activity (fig. S4, G and H). In addition, both the kinase-inactive and oxidized TNIK share a punctate localization in cells (movie S2 and fig. S1J).

### TNIK kinase activity is reversibly inhibited through H_2_O_2_ driven cysteine oxidation

**Fig. 7.**
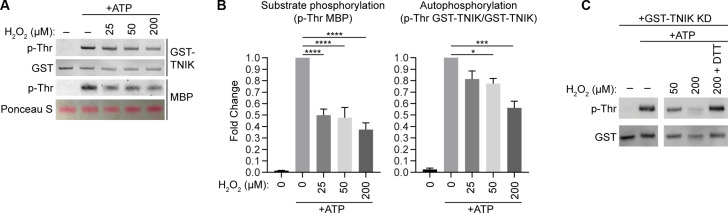
TNIK kinase activity is reversibly inhibited through cysteine oxidation by hydrogen peroxide. (**A**) GST-TNIK KD was mixed with MBP and subjected to in vitro kinase assays before Western blotting. Reaction mixtures were oxidized with the indicated concentration of hydrogen peroxide before the addition of ATP. (**B**) Quantification of (A), statistics was performed using a one-way ANOVA, means ± SEM, *P ≤ 0.05, ***P ≤ 0.001, and ****P ≤ 0.0001, *n* = 3 independent experiments. (**C**) GST-TNIK KD was oxidized with the indicated concentration of hydrogen peroxide, followed by reduction with DTT where indicated. In vitro kinase assays were then performed, followed by Western blotting.

We demonstrated that TNIK undergoes reversible oxidation of C202 after H_2_O_2_ treatment (Fig. 5). We performed in vitro kinase assays to gain a mechanistic understanding of the role of TNIK oxidation. Oxidation by H_2_O_2_ inhibited the ability of purified TNIK KD to autophosphorylate and to phosphorylate MBP substrate (Fig. 7, A and B). Moreover, oxidation of TNIK, followed by reduction with the thiol reductant DTT, restored TNIK kinase activity (Fig. 7C). This suggests that cysteine oxidation per se reversibly inhibits TNIK kinase activity and therefore exposes TNIK as a previously undiscovered redox sensor.

We next investigated the role of H_2_O_2_ oxidation on the TNIK-ERM pathway in the endothelium. Treatment of HUVEC with high amounts of H_2_O_2_ (1 to 2 mM) (*52*) resulted in a complete loss of ERM phosphorylation, without affecting total ERM levels (fig. S5A). In addition, we observed a faster migrating species of TNIK in SDS-PAGE upon treatment of HUVEC with 1 to 2 mM H_2_O_2_, consistent with TNIK deactivation and loss of autophosphorylation (fig. S5A). We predicted that the marked loss of active phosphorylated ERM upon high levels of H_2_O_2_ was due to terminal and irreversible cysteine oxidation of TNIK. Treatment of TNIK with 1 mM H_2_O_2_ resulted in a loss of kinase activity, which was only partially reversible upon treatment with DTT (compare fig. S5B with Fig. 7C). Although PEG-switch assays detected reversibly oxidized TNIK (Fig. 5, B to D), this technique cannot detect terminally oxidized cysteine thiols (*51*). To directly detect terminal cysteine oxidation, YFP-TNIK oxidized in vitro was subjected to mass spectrometry analysis. We detected trioxidation of TNIK C202 to a sulfonic acid group by mass spectrometry (fig. S5C). Our results suggest that TNIK acts as a redox sensor, which can be switched off through H_2_O_2_ oxidative signalling to modulate ERM activity.

### Blocking endogenous ROS in endothelial cells markedly increases p-ERM and promotes cell rounding in a KY-05009–sensitive manner

**Fig. 8.**
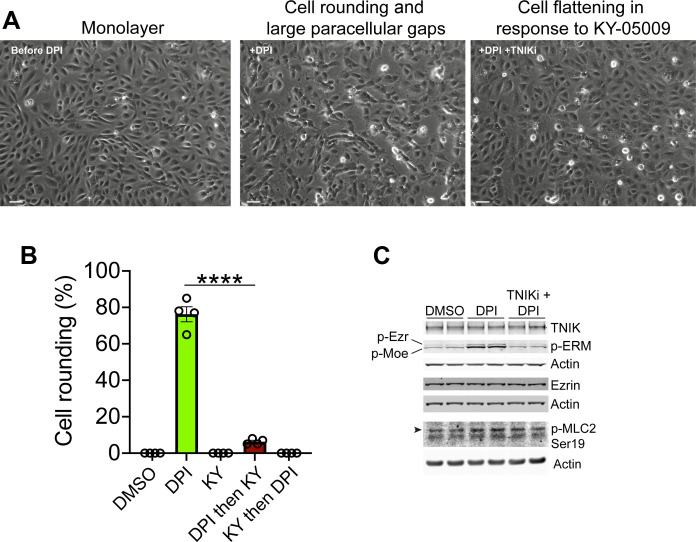
Blocking endogenous ROS in HUVEC induces cell rounding and large paracellular gaps in a KY-05009-sensitive manner. (**A**) HUVEC monolayer before and after treatment with DPI for 30 min (left and middle), followed by TNIK inhibition with KY-05009 for 2 hours (right). Stills from live-cell imaging time-lapse (see also movies S7 and S8). Scale bars 60 micrometers. (**B**) Quantification of cell rounding in HUVEC treated with DMSO (carrier), DPI, KY-05009 (KY), and DPI for 30 min and then KY for 2 hours or KY for 30 min and then DPI for 2 hours (see movie S9 for KY and then DPI). Statistical analysis using one-way ANOVA, means ± SEM, ****P ≤ 0.0001, four independent experiments. (**C**) Western blot of HUVEC whole cell lysates, representative of two independent experiments (one experiment resolved per lane). HUVEC treated with DMSO vehicle control, DPI or simultaneously with both DPI and KY-05009 (TNIKi, TNIK inhibitor) for 15 min followed by Western blotting. p-Ezr, p-Ezrin. p-Moe, p-Moesin. p-MLC2, phospho-myosin light chain at position serine 19. Arrowhead indicates the position of p-MLC2. Actin loading controls are for TNIK and p-ezrin, total ezrin, and p-MLC, respectively.

Overexpression of TNIK in epithelial cells can lead to cell rounding and detachment (*32*). Also, increases in p-ERM are causal to cell detachment, cell rounding, membrane tension, cortical rigidity, cell stiffness, and membrane blebbing in various cell types (*23*, *27*, *46*, *47*, *59*, *60*). We therefore asked, in a simple experimental setup, if suppressing endogenous ROS in HUVEC monolayers would lead to overactivation of TNIK and heightened ERM phosphorylation that would lead to cell rounding and detachment. Moreover, we asked whether suppression of constitutive endogenous ROS production was sufficient to induce cell rounding via a TNIK/ERM pathway. Using the pan-flavoenzyme inhibitor, diphenyleneiodonium chloride (DPI), was sufficient to induce plasma membrane blebbing and excessive endothelial cell retraction that promoted cell rounding and detachment, producing large paracellular gaps within 5 to 7 min (Fig. 8, A and B and movie S7). We also showed that challenging HUVEC first with DPI and then with KY-05009 reversed the DPI-induced gross endothelial cell retraction and blebbing phenotype (movie S8 and Fig. 8, A and B). We confirmed that the addition of DPI to cells was not a cytotoxic response by reversing the order in which the inhibitors were added: Preincubation of HUVEC with KY-05009 completely suppressed the cell blebbing and cell rounding phenotype in response to DPI addition (Fig. 8B and movie S9). We suspected that cell morphological changes to the endothelium upon ROS inhibition were due to excessive TNIK activity and ERM activation. Western blotting indeed confirmed that DPI treatment promoted strong increases in p-ERM levels, which was completely blocked in the presence of KY-05009 (Fig. 8C). We confirmed that these effects were independent of MLC2 phosphorylation (Fig. 8C). Together, these data suggest that TNIK activity is regulated by endogenous ROS-producing enzyme(s) and that its suppression can lead to rapid and marked increases in TNIK activity and ERM phosphorylation.

## DISCUSSION

TNIK is a redox sensor that plays an essential role in regulating TNF-α–induced endothelial permeability. A model is proposed whereby inflammatory TNF-α promotes phosphorylation and activation of endothelial ERM by TNIK, which anchors the plasma membrane to the cortical actin cytoskeleton during junctional instability, membrane retraction, and endothelial paracellular permeability, leading to inflammatory oedema formation and vascular leak. Furthermore, we show that TNF-α–induced paracellular gaps that form 7.5-hour post-stimulation can be resealed by the TNIK inhibitor, KY-05009, within 30 min. These data expose TNIK as a potential therapeutic target for suppressing hyperpermeability and excessive oedema, warranting further investigation.

TNF-α stimulation of HUVEC led to bimodal rises in ERM phosphorylation, peaking at 5 min and 8 hours (Fig. 4A). ECIS confirmed that TNF-α promoted permeability at the 4- and 8-hour time points (Fig. 2D). While the 4- and 8-hour time points corresponded well to paracellular gap formation (Fig. 3, A and B), little permeability was witnessed at the acute (5 min) time point (Fig. 2D). We believe that this period was too transient to observe any gross changes in permeability, likely due to the unstable formation and closure of paracellular gaps within an acute timescale. We did, however, witness transient increases in cellular stiffness (Fig. 4D) at timescales where TNF-α–induced HUVEC tension has been reported by traction force microscopy (*44*). Whether these observations are in vitro artifacts is yet to be determined. Cellular stiffness may be necessary for the fusion of secretory vesicles, e.g., Weibel-Palade bodies with the plasma membrane, to promote acute TNF-α–induced P-selectin expression for subsequent leukocyte tethering and rolling; however, this view is purely speculative and requires further investigation.

To our knowledge, there are no reports to suggest that TNF-α–induced permeability can occur at acute timescales. However, the methods we have used in this study are insufficiently sensitive to monitor these changes—particularly if accurate ECIS readings require periods of stabilization that fall into minutes after the addition of stimuli/treatments. Using a previously published cadaver model to investigate microvascular permeability of the cremasteric muscle (*61*), we did witness acute changes in permeability following acute exposure to TNF-α that was sensitive to KY-05009 treatment (fig. S6). However, these findings are too preliminary to make firm conclusions and will require further investigations beyond fluorescent albumin tracers, e.g., using electron dense tracers (like ferritin or horse radish peroxidase) to show venular leak between endothelial cells in rapidly fixed venules in TNF-α–treated but not control cremasteric preparations.

A notable observation was that very few paracellular gaps were observed at the 30-min time point (Fig. 3A), whereafter gaps would open progressively and consistently at the 4- and 8-hour time points. Moreover, the lack of paracellular gaps at the 30-min time point correlated with a significant decrease in p-ERM levels (compared to the 5-min and 8-hour time points; see Fig. 4A). We believe that this 30-min time point could reflect a period when TNIK is inactivated through oxidation, e.g. when endogenous ROS production is maximal. The source of ROS that is responsible for inactivating TNIK is currently not known, but isoforms of nicotinamide adenine dinucleotide phosphate oxidases (Nox), such as Nox2 and Nox5 (*62*), are activated by TNF-α (*63*) and calcium (*64*), respectively. In contrast, Nox4 produces H_2_O_2_ constitutively (*65*, *66*). Given the data presented in Fig. 8, it is tempting to speculate that Nox4 may be involved in constitutively suppressing TNIK activity. Other endogenous oxidases have not been formally excluded, mitochondrial (*67*) or extramitochondrial. More work is required to determine whether single or multiple oxidases are involved in regulating TNIK activity: before, during, and after TNF-α stimulation. In vivo, we cannot exclude the possibility that ROS could be derived from nonendothelial cells (e.g., neutrophils or perivascular macrophages) and contribute to paracrine regulation of endothelial TNIK in vivo. Under nonpathological conditions, resident perivascular macrophages that intimately contact the vasculature, maintain endothelial junctions, and suppress vascular permeability (*68*, *69*). It remains to be seen if ROS derived from perivascular macrophage (e.g., Nox2 or other oxidases) may signal directly to endothelial TNIK. Although H_2_O_2_ can diffuse through cellular membranes, potent intracellular reducing systems, such as peroxiredoxins, will greatly limit the effective range of H_2_O_2_ signaling (*52*). Thus, such a mechanism of paracrine ROS signalling would in principle allow specific regulation of plasma membrane–localized TNIK and ERM, without affecting other functions of TNIK such as nuclear transcriptional activation (*36*).

The small guanosine triphosphatase Rap2 coordinates the activation and membrane recruitment of TNIK and its paralogs MAP4K4 and MINK1, members of the MSN kinase subfamily (*70*–*74*). The siRNA depletion of Rap2 or MAP4K4 reduces endothelial permeability of unstimulated HUVEC, and knockdown of MAP4K4 also reduces endothelial permeability after overnight TNF-α stimulation (*75*, *76*). In contrast, siRNA knockdown of TNIK does not affect the permeability of unstimulated HUVEC nor thrombin-induced endothelial permeability (*75*, *77*). We find that siRNA depletion of TNIK, conditional knockout of endothelial-derived *Tnik*, or pharmacologic inhibition of TNIK reduces TNF-α stimulated, but not basal, endothelial permeability (Figs. 2 and 3). These results suggest a TNF-α–regulated function for TNIK in endothelial permeability and a constitutive role for MAP4K4. Similar to TNIK, MAP4K4 also phosphorylates ERM (*78*), and thus, the question remains how MSN kinases are differentially regulated. In contrast to MINK1 and MAP4K4, which are activated by H_2_O_2_ (*48*–*50*), we find that TNIK is inhibited by H_2_O_2_ oxidation and undergoes direct reversible oxidation of C202 (Figs. 5 to 7). It is unknown how MINK1 and MAP4K4 are activated by oxidative stress and whether this occurs directly. Although C202 is conserved between the MSN kinases, MINK1 has a unique C129, and MAP4K4 contains a unique C219, both within their KDs. Within the MAP4K4 structure (PDB: 4U3Y) C219 and C269 are positioned 4.9 Å apart, within the threshold for reversible disulfide bond formation (*54*). It would be interesting to investigate whether unique cysteine residues are responsible for the differential oxidative regulation of MSN kinases. Alternatively, binding partners unique to TNIK, perhaps regulated by the TNIK intermediate domain that is relatively less well conserved between TNIK homologs than the N-terminal KD (*32*), may control the differential redox regulation of TNIK and its homologs.

The constitutively kinase-dead K54A TNIK mutant can undergo reversible cysteine oxidation, and apposed C202 residues orientated to allow reversible disulfide bond formation are found in the inactive, but not the active, TNIK structure (Figs. 5G and 6, A and B, and fig. S4, G and H). As oxidation inhibits TNIK activity, it is likely that oxidation stabilizes the inactive TNIK conformation. On the basis of our experimental data, structural bioinformatics, MD/modeling, and known kinase activation mechanisms, we propose a mechanistic model for TNIK kinase activation and kinase inhibition by cysteine oxidation (Supplementary Discussion and fig. S7).

We identified that plasma membrane localized TNIK specifically drives ERM phosphorylation (Fig. 1J and fig. S1, H and I), which is likely to be distinct from other cellular functions of TNIK. For example, nuclear localized TNIK binds and phosphorylates the TCF4 transcription factor and thereby plays a crucial role in the activation of the Wnt signalling pathway (*36*). We have shown that only membrane-localized TNIK is susceptible to reversible cysteine oxidation (Fig. 5E). Why membrane-localized TNIK is sensitive to oxidation rather than cytosolic and nuclear TNIK is not known but may involve different TNIK binding partners or conformations that sterically hinder access to TNIK C202. Spatially controlled oxidation of TNIK may allow for localized regulation by membrane-associated ROS-generating enzymes and for control of ERM phosphorylation at endothelial junctions to affect permeability. Physical sequestration of TNIK away from the endothelial plasma membrane would provide a mechanism for regulating ERM function, as nonplasma membrane localized TNIK is unable promote ERM phosphorylation (Fig. 1J and fig. S1, H and J). In addition, we observe that kinase-dead K54A TNIK can be oxidized and localized largely to intracellular punctate compartments in endothelial cells, which may represent endosomes or peroxisomes (Fig. 5G and fig. S1J). Future studies will elucidate if TNIK oxidation, kinase activation status, and membrane trafficking involving signaling endosomes are interconnected to regulate TNIK signaling.

## MATERIALS AND METHODS

### Antibodies

Anti–p-ERM/anti–p-Dmoesin (Cell Signaling Technology, 3141), anti-GST (ChromoTek, 6G9), anti–p-Thr (Cell Signaling Technology, 9381), anti-GFP for Western blotting (ChromoTek, 3H9), anti-GFP for immunofluorescence detection of YFP-TNIK (Roche, 11814460001), anti-TNIK (Santa Cruz Biotechnology, sc-136103), anti-Actin (Sigma-Aldrich, A2228), anti–p-MLC2 Ser^19^ (Cell Signaling Technology, 3671), anti-Ezrin (Cell Signaling Technology, 3145), anti-moesin (Cell Signaling Technology, 3150), anti-ERM (Cell Signaling Technology, 3142), anti-vascular endothelial (VE)-cadherin for immunofluorescence (R&D Systems, goat polyclonal, AF938), anti–VE-cadherin for Western blotting (Santa Cruz Biotechnology, sc-6458), anti–glyceraldehyde-3-phosphate dehydrogenase (GAPDH) (Sigma-Aldrich, G9545), and anti–Histone H3 (Cell Signaling Technology, 9715).

### Cell culture of derived and primary cell lines

HUVECs from pooled donors (Lonza, Verviers Belgium or PromoCell, Heidelberg Germany) were cultured in Endothelial Growth Medium (EGM)-2 medium (Lonza) in a humidified incubator at 37°C in 5% CO_2_ up to four passages. For imaging or permeability assays, HUVECs were grown on substrate coated with bovine fibronectin (10 μg/ml; Sigma-Aldrich). More details on the permeability assays are provided below. For all other purposes, HUVECs were grown on substrate coated with 0.4% bovine gelatin solution (Sigma-Aldrich). HEK293T cells were cultured in Dulbecco’s modified Eagle’s medium (DMEM) supplemented with 10% fetal calf serum and 4 mM l-glutamine in a humidified incubator at 37°C in 5% CO_2_. To knockdown the expression of TNIK, HUVECs were transfected with control siGENOME RISC-Free (RF) siRNA (Dharmacon), TNIK #1 siRNA (Dharmacon, D-004542-07), or TNIK #2 siRNA (Dharmacon, D-004542-09) using Lipofectamine 2000 (Thermo Fisher Scientific), following the manufacturer’s instructions. To knockdown the expression of Nox2, HUVECs were transfected with ON-TARGETplus SMARTpool siRNA, Dharmacon. Assays were terminated 72 hours after siRNA transfection. Lipofectamine 2000 was used for transient plasmid transfection of cells according to the manufacturer’s instructions. DNA plasmids were used at a transfection mix concentration of 1 μg/ml. YFP-TNIK was a gift from J. Bos (University Medical Center, Utrecht, Netherlands). YFP-TNIK K54A, C108S, C134S, C202S, C234S, and C269S were generated using the Q5 Site-Directed Mutagenesis Kit (New England Biolabs) following the manufacturer’s instructions. To induce oxidative stress, HUVECs were washed three times with Hank’s balanced salt solution (HBSS; Sigma-Aldrich, H8264) and incubated with the indicated time and concentration of H_2_O_2_ in HBSS. Unless otherwise stated in the figure legends, HUVECs were treated for the indicated times with: TNF-α (10 ng/ml; Bio-Techne, 8599-TA-010), 10 μM KY-05009 (Sigma-Aldrich), 50 μM PF-794 (AOBIOUS), 10 μM NCB-0846 (MedChemExpress), 10 μM DPI (Sigma-Aldrich).

Pulmonary microvascular endothelial cells were affinity selected using magnetic beads (CD31 microbeads, mouse, Miltenyi Biotec). Lungs were isolated from euthanized mice and washed in phosphate-buffered saline (PBS) containing 1% of penicillin/streptomycin (100 U/ml; Sigma-Aldrich). Lungs were finely minced using surgical microscissors and scalpel and subsequently subjected to enzymatic digestion with collagenase/dispase (Merck) diluted 1 mg/ml in DMEM (Corning) for 45 min at 37°C with continuous shaking. Tissue suspension was then passed through 18-gauge needle 12 times and serially filtered through 70- and 30-μm cell strainers. The cells obtained were then incubated with CD31 MicroBeads (Miltenyi Biotec) following the manufacturer’s instructions. CD31-positive cell fraction was centrifuged and washed in PBS before DNA extraction and polymerase chain reaction (PCR) analysis of exon 6. Genomic DNA extraction was performed using NaOH 50 mM and 1 M tris-Cl (pH 8) solutions as described in the section below.

### Mouse strains

All mice were maintained in a climatically controlled environment and had access to food and water ad libitum. All experiments were carried out in accordance with the 1986 UK Home Office Animals (Scientific Procedures) and UK Arrive guidelines, with ethics approval by King’s College London. All studies with mice were conducted under the project license number PA93DA655. Male CD1 mice weighing 25 to 35 g (Charles River Laboratories, strain code: 022) were used for skin plasma extravasation assays with the TNIK inhibitor NCB-0846. For endothelial-specific tamoxifen-inducible *Tnik* knockout study, Tniktm3a(EUCOMM)Wtsi female mice, carrying *Tnik* floxed at exon 6 (i.e. homozygous and flanked by loxP sites) in chromosome 3, were crossed to male Tg(Cdh5-cre/ERT2)1Rha (*79*) mice to obtain tamoxifen-inducible Cre-expressing *Tnik* conditional knockout mice—specifically in endothelial cells. Cre-negative, *Tnik*-floxed littermates were used as control mice and were injected with tamoxifen (see below for more details). Mice were between the ages of 8 to 12 weeks (adults).

### Western blotting

Cells were lysed directly in sample buffer [100 mM tris-HCl (pH 6.8), 4% (w/v) SDS, 20% (w/v) glycerol, 200 mM DTT, and 0.005% (w/v) bromophenol blue] supplemented with benzonase nuclease (100 U/ml; Sigma-Aldrich) and incubated for 5 min at room temperature, followed by boiling for 5 min and resolving on SDS-PAGE gels (Invitrogen). For nonreducing SDS-PAGE, proteins were boiled in sample buffer without DTT. To resolve oxidised and reduced forms of TNIK, proteins were electrophoresed on Novex 6% tris-glycine gels. To resolve Ezrin and Moesin, proteins were electrophoresed on NuPAGE 3 to 8% tris-acetate gels. All other samples were electrophoresed on Bolt 4 to 12% bis-tris Plus gels. Resolved proteins were transferred onto nitrocellulose membranes (Amersham). Membranes were blocked in 5% (w/v) milk powder dissolved in tris-buffered saline, followed by incubation with primary and secondary antibodies. Blots were visualized by enhanced chemiluminescence (ECL Prime) detected with photographic film developed using an automatic processor. For quantitative blotting, blots were visualized using IRDye LI-COR secondary antibodies and the Odyssey CLx Near-Infrared Fluorescence Imaging System, followed by quantitation using Image Studio (LI-COR) software.

### Drosophila kinome-wide RNAi screen

Screening was performed at the Wellcome Trust Sheffield RNAi Screening Facility (*80*). *D. melanogaster* S2R^+^ cells were treated with a kinome collection of 843 dsRNAs targeting 425 kinases in 3 × 384–well plates. Library design is based on the HD2.0 RNAi library (Heidelberg 2) (*81*). Three days after, knockdown cells were fixed in 10% trichloroacetic acid in PBS for 10 min on ice and were then permeabilized in 0.1% NP-40 for 3 min on ice. Cells were blocked in 5% bovine serum albumin (BSA) in PBS for 1 hour at room temperature. Cells were then incubated with primary antibody (1:300; anti–p-ERM, Cell Signaling Technology, 3141) in 5% BSA in PBS overnight at 4°C. Cells were labeled with secondary antibody conjugated to Alexa Fluor 488 and 4′,6-diamidino-2-phenylindole (DAPI) to stain nuclei. Imaging was performed using the ImageXpress Micro Confocal High-Content Imaging System (Molecular Devices), and images were analyzed using MetaXpress software. For each dsRNA, a score was assigned (arbitrary scale) based on the ratio of non-nuclear/nuclear p-Dmoesin staining. After quality control and manual curation of images, 414 kinases were taken forward for analysis. Data analysis was performed using R3.6.2 (www.R-project.org).

### Immunoprecipitation

HEK293T cells were transfected with the indicated constructs and 24-hour post-transfection cells were lysed using Pierce IP Lysis buffer [25 mM tris-HCl (pH 7.4), 150 mM NaCl, 1% NP-40, 1 mM EDTA, and 5% glycerol] supplemented with PhosSTOP (Roche) and Pierce Protease Inhibitor, EDTA free. Lysates were clarified by centrifugation, and YFP-tagged proteins were immunoprecipitated using GFP-Trap beads for 1 hour at 4°C. Immunoprecipitates were stringently washed three times in Pierce IP lysis buffer containing 500 mM NaCl and subjected to in vitro oxidation with H_2_O_2_ or used for in vitro kinase assays. Last, immunoprecipitates were eluted in sample buffer [100 mM tris-HCl (pH 6.8), 4% (w/v) SDS, 20% (w/v) glycerol, 0.005% (w/v) bromophenol blue] supplemented with or without 200 mM DTT for reducing or nonreducing SDS-PAGE, respectively, by boiling for 5 min, followed by SDS-PAGE and Western blotting.

### GST-tagged protein expression

GST or GST-Moesin C-terminal domain was expressed in *E. coli* JM109 cells (Sigma-Aldrich) in LB medium. GST-Moesin C-terminal domain was a gift from A. Bretscher (Weill Institute for Cell and Molecular Biology, Cornell University, USA). Expression was induced by addition of 1 mM isopropyl-β-D-thiogalactopyranoside at optical density at 600 = 0.6, and cells were incubated at 37°C for 4 hours. Harvested cells were lysed using sonication on ice in a lysis buffer (PBS + 1% Triton X-100, supplemented with Pierce Protease Inhibitor), and the clarified supernatant was subsequently applied to Glutathione Sepharose 4B beads (GE Healthcare). After several washes with (PBS +1% Triton X-100 + 500 mM NaCl supplemented with Pierce Protease Inhibitor), fusion protein-bound beads were used directly for in vitro kinase assays or subjected to SDS-PAGE and Coomassie G-250 gel staining (SimplyBlue Safe Stain, Thermo Fisher Scientific).

### In vitro kinase assays

Recombinant purified GST-TNIK KD 200 ng of human amino acids 1 to 367 (Thermo Fisher Scientific) or YFP-TNIK expressed in HEK293T cells and immobilized on GFP-TRAP beads by immunoprecipitation (as described above) were mixed with substrate, 1 μg of MBP (bovine, Sigma-Aldrich) or GST-Moesin C-terminal domain immobilized on Glutathione Sepharose 4B beads in kinase buffer [25 mM tris-HCl (pH 7.4), 10 mM MgCl_2_, and 5 mM β-glycerophosphate). Where indicated, reaction mixtures were oxidized with the indicated concentration of H_2_O_2_ for 15 min at 30°C, where indicated reactions where then reduced with 50 mM DTT for 30 min at 30°C. Kinase reactions were then performed by the addition of 200 μM ATP at 30°C for 30 min. Reaction products were subjected to SDS-PAGE and Western blotting.

### Tamoxifen injection protocol

Male and female mice aged between 12 and 15 weeks received 100 μl of tamoxifen-dissolved peanut oil (final concentration: 20 mg/ml) via intraperitoneal injection for three consecutive days. This dose is equivalent to 100 mg/kg body weight per day for a 20-g mouse. Skin plasma extravasation assays followed 7 days after the last tamoxifen injection. The efficiency of *Tnik* knockout was assessed 10 days after the first tamoxifen injection, PCR using primers design to amplify *a* ≈ 1300–base pair (bp) amplicon in Cre-negative mice and ≈400 bp amplicon in Cre-positive recombinant mice (indicating the deletion of exon 6; see table S2). *Tnik* knockout was observed to be 50% from skin biopsies due to amplification of exon 6 from stromal (nonendothelial) cells (fig. S3D). In contrast, 100% efficiency of knockout was observed in pulmonary microvascular endothelial cells (fig. S3E), isolated from tamoxifen-injected Cre-positive (but not Cre-negative) mice.

### PEG-switch assay

Reversible protein oxidation was assessed as previously described using the PEG-switch assay (*51*). Briefly, HUVECs were washed three times with HBSS (Sigma-Aldrich, H8264) and incubated with the indicated time and concentration of H_2_O_2_ in HBSS. HUVECs were lysed into alkylating buffer [1% SDS, 100 mM maleimide, and 100 mM tris-HCl (pH 7.4)] and then heated at 50°C for 25 min. Alternatively, YFP-TNIK immobilized on GFP-Trap beads and oxidized in vitro with H_2_O_2_ were used for PEG-switch assays. Alkylated samples were then supplemented with 200 mM DTT to quench the maleimide and reduce reversibly oxidised thiols. After 30-min incubation, each sample was desalted (Zeba columns, Thermo Fisher Scientific) and eluted into labeling buffer [10 mM PEG-maleimide, 5 kDa (Sigma-Aldrich, 63187), 1% SDS, and 100 mM tris-HCl (pH 7.4)]. For YFP-TNIK on beads, samples were washed into labeling buffer. Following 2 hours further incubation at room temperature, samples were prepared for SDS-PAGE by the addition of sample buffer containing DTT and Western blotted as described above.

### Subcellular fractionation

HUVECs were washed three times with HBSS (Sigma-Aldrich, H8264) and incubated with the indicated time and concentration of H_2_O_2_ in HBSS. HUVECs were scraped into ice-cold Hepes buffer [20 mM Hepes-KOH (pH 7.5), 250 mM sucrose, 10 mM KCl, 2.5 mM magnesium acetate, 1 mM EDTA, PhosSTOP (Roche), Pierce Protease Inhibitor EDTA-free, and 100 mM maleimide] and then homogenized by passing through a 27-gauge hypodermic needle. Homogenization was monitored by Trypan Blue staining. Nuclei were removed by centrifugation at 5000 rpm for 5 min at 4°C in an Eppendorf micro centrifuge, and this was then repeated. The post-nuclear supernatant was subjected to centrifugation at 112,500*g* for 1 hour at 4°C to obtain the membrane pellet and the cytosol supernatant. Where indicated, membrane pellets were resuspended in Hepes buffer supplemented with 500 mM NaCl, and the suspension was subjected to centrifugation at 112,500*g* for 1 hour at 4°C to obtain proteins tightly associated with the membrane. Where indicated, membrane pellets were resuspended in Hepes buffer supplemented with 1% Triton X-100, and the suspension was subjected to centrifugation at 112,500*g* for 1 hour at 4°C to obtain proteins associated with detergent resistant membranes. Membrane pellets were then solubilized in alkylating buffer [1% SDS, 100 mM maleimide, and 100 mM tris-HCl (pH 7.4)]. Nuclear pellets were solubilized in alkylating buffer [1% SDS, 100 mM maleimide, and 100 mM tris-HCl (pH 7.4)] supplemented with benzonase nuclease (100 U/ml; Sigma-Aldrich). The 1% SDS was added to the post-nuclear supernatant and cytosol fractions. All fractions were then subjected to PEG-switch assay, SDS-PAGE, and Western blotting as detailed above. Membrane fractions are loaded on SDS-PAGE gels at 5× concentration compared with nuclear, cytosol, and post-nuclear supernatant fractions to aid with comparison.

### Mass spectrometry: Sample preparation, LC-MS/MS analysis, and database searching

#### *Mass spectrometry*—*Sample preparation*

YFP-TNIK was expressed in HEK293T cells and immunoprecipitated using GFP-Trap beads as described above. Immunoprecipitated YFP-TNIK was oxidized in vitro using 200 μM H_2_O_2_ for 15 min at 30°C. Immunoprecipitates were eluted into sample buffer [100 mM tris-HCl (pH 6.8), 4% (w/v) SDS, 20% (w/v) glycerol, 200 mM DTT, and 0.005% (w/v) bromophenol blue] by boiling for 5 min followed by SDS-PAGE as described above and Coomassie G-250 gel staining (SimplyBlue Safe Stain, Thermo Fisher Scientific). The YFP-TNIK band was excised and subjected to digestion. To preserve cysteine modifications on the protein, no reduction or alkylation steps were performed. YFP-TNIK was digested into peptides using trypsin (bovine, Sigma-Aldrich) in a 1:5 ratio (enzyme/substrate) incubated in a shaking heat block at 37°C for 16 hours at 750 rpm. YFP-TNIK was further digested with endoproteinase Asp-N (1:5 ratio; Sigma-Aldrich). Peptides were extracted in a series of solvent and aqueous dehydration/hydration steps using acetonitrile (Thermo Fisher Scientific, UK) and 50 mM triethylammonium bicarbonate (Sigma-Aldrich) and pooled. Peptide supernatant was dried to completion by SpeedVac (Thermo Fisher Scientific, UK) for 2 hours (9000 rpm, 45°C).

#### *Mass spectrometry*—*LC-MS/MS analysis*

The peptide sample was resuspended in 2% acetonitrile in 0.05% formic acid (both Thermo Fisher Scientific, UK) to be analyzed by liquid chromatography tandem mass spectrometery (LC-MS/MS). Chromatographic separation was performed using a U3000 UHPLC NanoLC system (Thermo Fisher Scientific, UK). Peptides were resolved by reversed phase chromatography on a 75-μm C18 Pepmap column (50 cm in length) using a three-step linear gradient of 80% acetonitrile in 0.1% formic acid. The gradient was delivered to elute the peptides at a flow rate of 250 nl/min over 60 min starting at 5% B (0 to 5 min) and increasing solvent to 40% B (5 to 40 min) before a wash step at 99% B (40 to 45 minutes) followed by an equilibration step at 5% B (45 to 60 minutes).

The eluate was ionized by electrospray ionization using an Orbitrap Fusion Lumos (Thermo Fisher Scientific, UK) operating under Xcalibur v4.1.5. The instrument was first programmed to acquire using an Orbitrap-Ion Trap method by defining a 3-s cycle time between a full MS scan and MS/MS fragmentation. Orbitrap spectra (FTMS1) were collected at a resolution of 120,000 over a scan range of mass/charge ratio (*m*/*z*) 375 to 1500 with an automatic gain control (AGC) setting of 4.0 × 10^5^ with a maximum injection time of 35 ms. Monoisotopic precursor ions were filtered using charge state (+2 to +7) with an intensity threshold set between 5.0 × 10^3^ to 1.0 × 10^20^ and a dynamic exclusion window of 35 s ± 10 parts per million (ppm). MS2 precursor ions were isolated in the quadrupole set to a mass width filter of 1.2 *m*/*z*. Ion trap fragmentation spectra (ITMS2) were collected with an AGC target setting of 1.0 × 10^4^ with a maximum injection time of 35 ms with collision-induced dissociation collision energy set at 35%. This method takes advantage of multiple analyzers on Orbitrap Fusion Lumos and drives the system to use all available parallelizable time, resulting in decreasing the dependence on method parameters.

#### *Mass spectrometry*—*Database searching*

Raw data files were processed using Proteome Discoverer (v2.2, Thermo Fisher Scientific) to search against the sequence of TNIK protein (UniProt accession number Q9UKE5) with Mascot search algorithm (v2.6.0, www.matrixscience.com) and the Sequest search algorithm (*82*). Precursor mass tolerance was set to 20 ppm with fragment mass tolerance set to 0.8 Da with a maximum of two missed cleavages. Variable modifications included: oxidation (Met), dioxidation (Cys), and trioxidation (Cys). Database generated files (.msf) were uploaded in to Scaffold software (v 4.10.0, www.proteomesoftware.com) for visualization of fragmentation spectra and PTM detection.

### MD simulation systems and parameters

#### System setup

The atomic coordinates of the crystal structure of TNIK in inactive conformation were retrieved from the protein data bank (PDB entry: 5CWZ, resolution: 2.9 Å). The structure is in a trimeric form, only two chains were kept (chain A and B). The regions encompassing residues 175–185 in 5CWZ, not solved in the crystal structure, were modelled using the SWISS-MODEL webserver (*83*). Rigid protein docking was performed with the ClusPro 2.0 webserver (*84*) to produce a starting structure of the dimer with the C202 residues (in adjacent TNIK KD monomers) in closer proximity. The server generated a docked structure in which the distance of C202 residues was 5.3 Å apart; in the 5CWZ structure C202 residues are 11.8 Å apart. The crystal structure and the docked structure are highly superimposable with an RMSD of 3.3 Å, hence, we proceeded with the docked model for further analysis. This model is termed: WT TNIK docked (inactive). To produce a mutant TNIK model with C202S in both monomers of the dimer, in silico mutagenesis in the wt-TNIK docked model was performed with the DUET webserver (*85*). This model is termed mt-TNIK docked (inactive). The treated structures were used as the initial structures for MD simulations.

### MD simulations

MD simulations of human TNIK were performed with the GROMACS software version 2019.3 (www.gromacs.org/) and were carried out in explicit water using Amber ff14SB force field (*86*). MD simulations were performed in the WT-TNIK crystal structure (PDB:5CWZ), WT-TNIK docked (inactive) model, and mt-TNIK docked (inactive) model using the same parameters. The proteins were solvated in a cubic box with a periodic boundary margin distance of 20.0 Å in TIP3P water (*87*). The system charge was neutralized by adding counter ions to solvent (Na^+^ and Cl^−^). The systems were energy minimized for 2.000 steps with positional restraints in the heavy atoms using steepest descent algorithm (step size of 0.01 nm and tolerance of 100 kJ mol^−1^ nm^−1^) and then for another 10.000 steps without any restraints. The systems were equilibrated in the NVT ensemble with decreasing positional restraints at increasing temperatures in four stages of 100 ps each: at 50, 100, 200, and 300 K with force constant restraints of 2000, 1000, 500, and 250 kJ/(mol nm^2^), respectively. This was followed by a further equilibration in the NPT ensemble where the systems were gradually heated at 100, 200, and 300 K without restraints for 100 ps each, with the velocity rescale thermostat and 1 bar (Berendsen barostat) (*88*, *89*). Following equilibration, multiple, independent, replicate production MD simulations of 50 ns-long were performed under constant pressure and temperature conditions, 1 bar and 300 K. The Parrinello-Rahman barostat (*90*) with isotropic coupling and velocity rescale thermostat with a coupling constant of 0.1 ps^−1^ were implemented to maintain constant pressure and temperature, respectively. During the MD simulations, the long-range Coulombic interactions were treated using the particle-mesh Ewald (PME) method (*91*). Bond lengths of heavy atoms were constrained using the Linear Constraint Solver (LINCS) algorithm (*92*). The cutoff distance for van der Waals and Coulomb energy terms was set at 12.0 Å.

Analysis of MD trajectories (RMSD, distances, and COM) were performed using GROMACS tools. The concatenated trajectories containing all four four simulations both for the mt-TNIK–docked and WT-TNIK–docked systems were analyzed using Python language (www.python.org). PyMOL (The PyMOL Molecular Graphics System, version 2.3.5 Schrödinger, LLC) and Visual Molecular Dynamics package (*93*) were used for visualization of trajectories and rendering of molecular model illustrations.

### Protein interface analysis

PDBePISA (protein interfaces, surfaces, and assemblies) (www.ebi.ac.uk/pdbe/pisa/) (*56*) and POPSCOMP (*94*) were used to analyze the protein-protein interaction interface of the TNIK crystal structure (5CWZ) and WT-TNIK–docked (inactive) model. POPSCOMP was used to determine the individual residues contribution to either the hydrophobicity or hydrophilicity of the interface determined by their solvent accessible surface area; the algorithm was run using default parameters (*94*).

### Measurement of paracellular gaps

Endothelial gaps were quantified from immunofluorescence images by converting signals from DAPI, p-ERM, and VE-cadherin channels to grayscale, setting gamma to 1.5 to enhance low-level contrast and then using the threshold and analyze particles functions on ImageJ to identify regions with low signal (gaps, where no parts of cells are overlying the coverglass). Graphed data are means of four images from each time point for each batch of HUVEC. Wider field images can be seen in fig. S2 (A and B).

### Endothelial transwell permeability assay

HUVEC or HUVEC-transfected with RISC-Free (RF) control siRNA or TNIK #2 siRNA was seeded onto transwell inserts (12 mm in diameter, 0.4-μm pore size, polyester membrane; Corning) coated with bovine fibronectin (10 μg/ml; Sigma-Aldrich) and stimulated with TNF-α (10 ng/ml), after forming a confluent monolayer. Four hours after TNF stimulation, 3 μg of tetramethyl rhodamine isothiocyanate (TRITC)–BSA (67 kDa), 3 μg of fluorescein isothiocyanate (FITC)–dextran (4 kDa) and (where indicated) dimethyl sulfoxide (DMSO) vehicle control or 10 μM KY-05009 TNIK inhibitor added to the upper chamber. One hour after the addition of fluorescent tracers, 100 μl of aliquots was taken from the lower chambers and transferred into 96-well black, opaque-bottomed, Nunclon plates, and the fluorescence intensity was measured using a Tecan Infinite M200 PRO plate reader.

### Electrical cell-substrate impedance sensing

HUVECs were purchased from Lonza and PromoCell (two donors, passages 2 to 5) and grown on fibronectin-coated culture flasks. Cells were cultured in complete ECIS (ScienCell Research Laboratories) at 37°C and 5% CO_2_, with a medium change every other day. To measure the effect of TNIK inhibition on endothelial barrier resistance, we used ECIS: cells were seeded in 96W10idf 96-well plates (IBIDI) using 1.5 × 10^4^ cells per well, with a medium change 24 hours after seeding. Following 48 hours after seeding, HUVECs were pretreated with KY-05009 at 10 μM final concentration, dissolved in DMSO (0.1% final concentration), or with 0.1% DMSO for 2 hours. Subsequently, cells were stimulated with TNF-α (10 ng/ml in final concentration, R&D Systems). For all experiments, endothelial resistance was measured at 4000 Hz with an interval time of 2 min.

### Quantification of paracellular gaps in HUVEC

Endothelial gaps were quantified from immunofluorescence images by converting signals from DAPI, p-ERM, and VE-cadherin channels to grayscale, setting gamma to 1.5 to enhance low-level contrast and then using the threshold and analyze particles functions on ImageJ to identify regions with low signal (gaps, where no parts of cells are overlying the coverglass). Graphed data are means of four images from each time point for each batch of HUVECs.

### Mechano scanning ion conductance microscopy

The mechanoSICM was used to simultaneously generate topography and transverse Young’s modulus (YM) maps (100 μm by 100 μm). A positive pressure of 15 kPa was delivered to the nanopipette and applied constantly while scanning. The nanopipettes (200- to 300-nm tip diameter) were pulled from borosilicate capillary glass using a laser puller (Sutter Instrument Co. P-2000). The aerostatic pressure that propels the inner pipette solution forms a hydrojet that displaces the cell’s plasma membrane and records this change in displacement. Three setpoints of the current-distance dependence were selected, one to map the topography (0.7% of current reduction) and two (SP1 and SP2) to map the YM (1 and 2% current reduction, respectively). From the changes observed between SP1 and SP2, the YM is calculated, as described previously (*95*). PromoCell complete HUVEC growth medium, supplemented with 25 mM Hepes, was used for the bath and pipette solution. Images were analyzed using SICM Image Viewer.

### TNIK genotyping

Mice were genotyped through a combination of separate PCR reactions that specifically detects the WT TNIK allele and the floxed mutant TNIK allele (at exon 6), the Cre recombinase transgene and GAPDH as internal control. To obtain genomic DNA, mouse ear biopsies were boiled for 30 min in 300 μl of NaOH 50 mM solution. After boiling, the solution was neutralized by adding 50 μl of 1 M tris-Cl (pH 8). PCR was performed using primer pairs listed in table S3. A typical 25 μl pf PCR reaction mix contains 12.5 μl of Taq DNA Polymerase (GoTaq Green Master Mix, Promega), 0.5 μM forward and reverse primer pairs, and 5 μl of genomic DNA template obtained from ear biopsies. The standard PCR condition was as follows: TNIK WT/Floxed: 94°C for 5 min, 94°C for 30 s, 58°C for 30 s, and 72°C 45 s for 35 cycles; Cre/GAPDH 94°C for 5 min, 94°C for 30 s, 60°C for 30 s, and 72°C 1 min for 35 cycles. PCR products were resolved with 2.5% tris, acetate, and EDTA containing Nancy-520 (0.375 ng/μl).

### Skin plasma extravasation assays (also known as “Miles assay”)

Skin plasma extravasation was determined by the extravascular accumulation of Evans blue adapted from Sawyer *et al.* (*96*), and more recently by Zarban *et al.* (*40*). TNIK inhibitor NCB-0846 (80 mg/kg) in suspension in 5% DMSO or DMSO vehicle control was administered by intraperitoneal injection in CD1 mice. The dorsal skin of the animals was shaved and prepared for intradermal injection (six sites per mouse, each in a randomly allocated symmetrical site pattern). Thirty min after administration of TNIK inhibitor, Evans blue (1.25%; Sigma-Aldrich) was injected intravenously into the lateral tail vein (50 μl/10 g body weight) 5 min before i.d. treatments. After 5 min, 50 μl of vehicle (Tyrode’s solution: 137 mM NaCl, 2.68 mM KCl, 0.42 mM NaH_2_PO_4_, 11.9 mM NaHCO_3_, 1.05 mM MgCl_2_, and 5.55 mM glucose in distilled water), murine recombinant TNF-α (R&D Systems), zymosan, injection only control (Sham) were injected intradermally into the dorsal skin. Animals were left for 4 hours to allow plasma extravasation to occur. At the end of the accumulation period, the mice were euthanized by cervical dislocation, and the dorsal skin was removed. Oedema volume was measured at intradermal injection sites where plasma extravasation of Evans blue had occurred. Two perpendicular intersecting diameters across the extravasation area were measured, and the skin thickness was measured using a micrometer. The volume of an ellipsoid was calculated to give the oedema volume measurement.

### MPO assay

MPO assay was adapted in (*97*) and is as described in (*40*). Skin sites were prepared as described earlier, homogenized in 500 μl of homogenization buffer [0.1 M sodium chloride, 0.02 M sodium phosphate, 0.015 M EDTA, and 0.5% hexadecyl trimethyl ammonium bromide (pH 4.7), all reagents are from Sigma-Aldrich, St. Louis, MO], and lyzed using QIAGEN TissueLyser II (Thermo Fisher Scientific, Warrington, UK) at 30 Hz for 5 min, three times, with cooling in between. Samples were centrifuged at 17,000*g* for 15 min at 4°C, and the supernatant was collected. Reactions were performed in a 96-well plate at room temperature. Hydrogen peroxide oxidation of TMB Liquid Substrate System (TMB, Sigma-Aldrich, St. Louis, MO) was used to determine MPO activity. In a 96-well plate, 25 μl of MPO buffer was added to 25 μl of the sample, and 100 μl of TMB liquid substrate was then added. The plate was incubated in the dark at 37°C for 15 min. Absorbance (OD) was read at 620 nm using a spectrophotometer, and a standard curve of OD against MPO in the standard samples was plotted. In some cases, protein (BSA) concentrations were also measured by adding 25 μl of alkaline copper tartrate solution (Bio-Rad Laboratories, Watford, UK) to 1 μl of the sample (diluted 1:5 in homogenization buffer), and 200 μl of dilute Folin (Bio-Rad Laboratories, Watford, UK) was then added. OD was read at 700 nm, thus acquiring the quantification of MPO/protein in each condition.

### Confocal microscopy

For p-ERM staining, HUVECs transiently transfected with the indicated constructs and grown on coverslips were fixed in 10% trichloroacetic acid in PBS for 15 min on ice. Following fixation, HUVECs were permeabilized in 0.1% NP-40 substitute for 3 min. Next, coverslips were blocked in 33% fetal calf serum in PBS for 30 min followed by incubation with primary antibody in 33% fetal calf serum in PBS overnight at 4°C. Following this, coverslips were washed in PBS and incubated with secondary antibody in the same buffer as primary for 1 hour at room temperature before final washing with PBS and water. Coverslips mounted on slides were imaged using either a Nikon Spinning Disk confocal system (Nikon Eclipse TiE equipped with a CSU-X unit and an Andor iXON Ultra 897) at the Wohl Cellular Imaging Centre at King’s College London. Images were acquired using a 20× air or 60× oil immersion objective [numerical aperture (NA) 1.4]. Images from fig. S2 (A and B) and Fig. 3 (B and E) and 4B used an Andor BC43 benchtop microscope, 40× air lens (NA 0.95). Anti-GFP (to detect YFP-TNIK) and anti–VE-cadherin were labeled with secondary antibodies conjugated to Alexa Fluor 488 and excited with a 488-nm diode laser. Anti–p-ERM was labeled with secondary antibody conjugated to Alexa Fluor 568 and excited with a 561-nm diode laser. F-actin was labeled TRITC-phalloidin and excited using a 561-nm diode laser. To generate a 3D isometric view of p-ERM–positive plasma membrane blebs, images were taken as a series of Z-stacks followed by volumetric rendering in NIS elements software (Nikon).

### Live-cell imaging

Live-cell imaging was performed on HUVEC or HUVEC transduced with TD Moesin-GFP lentivirus (where TD moesin represents threonine-558 in moesin replaced with aspartate to mimic constitutive phosphorylation) (*98*). During live-cell imaging, HUVECs were maintained at 37°C in EGM-2 buffered with 25 mM Hepes. For fluorescent imaging, HUVECs were imaged on chambered coverslips (ibidi) using a Nikon Spinning Disk confocal system (Nikon Eclipse TiE equipped with a CSU-X unit and an Andor iXON Ultra 897) at the Wohl Cellular Imaging Centre at King’s College London. Images were acquired using a 60× oil immersion objective (NA 1.4). TD Moesin-GFP was excited with a 488-nm diode laser.

For cell rounding experiment, bright-field live-cell imaging of HUVEC was performed on an inverted Olympus IX-81 microscope with a 10× air objective. DPI (10 μM) was added to confluent endothelial monolayers to induce paracellular gap formation, followed by the addition of 10 μM KY-05009 TNIK inhibitor. Alternatively, 10 μM KY-05009 or 50 μM PF-794 TNIK inhibitor was added simultaneously with DPI treatment.

### Microvascular permeability experiments

The technique has been previously described extensively (*61*). Briefly, male mice (CD1 20 to 40 g, Charles River Laboratories, UK), were euthanized by exposure to a rising concentration of CO_2_, followed by cervical dislocation. A longitudinal midline incision (1 to 2 cm) was made along the abdomen to expose the underlying organs, the aorta cannulated orthogradely, and the vena cava punctured to create an outlet for the blood that was flushed out of the circulation. The lower portion of the mice was perfused with a modified St. Thomas’ cardioplegic solution (34 mM MgCl_2_, 110 mM NaCl, 8 mM KCl, 1 mM CaCl_2_, and 11 mM Hepes) containing heparin (30 U/ml) and 10 μM isoproterenol buffered to pH 7.0 ± 0.05 for 10 min or until the effluent was discernibly clear of blood (*99*).

### Cremaster muscle superfusion

The stabilizing solution perfusing the cremaster vasculature was replaced with Krebs solution (124 mM NaCl, 4.7 mM KCl, 2.5 mM CaCl_2_, 1.18 mM MgSO_4_·7H_2_O, 1.2 mM KH_2_PO_4_, 25 mM NaHCO_3_, 10 mM glucose, and 11 mM Hepes and buffered to pH 7.4 ± 0.05) containing bovine albumin (10 ml/min) delivered via a pressure regulated rotary pump with the temperature controlled at 37°C at a rate of 1.0 ml/min. After 30 min, Krebs perfusion of the vasculature was stopped, and a bolus of Krebs solution containing FITC-conjugated albumin (5 mg/ml) was injected into the perfusion line. Post-capillary venules were identified by noting the direction of flow, as the microvasculature was filled with the fluorescent dye using a Zeiss ACM microscope 20× water immersion objective (numerical aperture 0.5). Images were captured using a FITC filter cube (Chroma Technology, Bellows Falls, VT, USA) via image-intensified charge-coupled device camera (Photonic Sciences, Robertsbridge, E. Sussex, UK) for subsequent analysis (ImageHopper, Samsara Research, Dorking, Surrey, UK). The perfusion pressure was lowered to allow pressure differences within the vasculature to dissipate.

When required TNF-α and KY-05009 were included in the superfusion fluid to give final concentrations of 10 ng/ml and 10 μM, respectively. Permeability measurements were obtained from an image sequence acquired at 1-s intervals. The dye concentration difference across a vessel was calculated from the difference between the mean pixel values in the regions of interest positioned on an image stack (see fig. S6). Permeability (*P*) was determined from the rate of decrease in that difference, obtained by fitting an exponential to the data (fig. S6), such that *P* = *kD*/4, where *k* is the rate constant and *D* is the vessel diameter.

### Statistical analysis

The statistical details of all experiments are reported in the figure legends and figures, including statistical analysis performed, error bars, statistical significance, and *n* numbers. Statistics were performed using GraphPad Prism 8 software, as detailed in all the figure legends.

## Supplementary Materials

### The PDF file includes:

Supplementary Discussion Figs. S1 to S7 Tables S2 and S3 Legend for table S1 Legends for movies S1 to S9 References
