# Supplementary material for: TNIK: A redox sensor in endothelial cell permeability
Source: Sci Adv. 2024 Dec 20;10(51):eadk6583. doi: 10.1126/sciadv.adk6583 (PMC11661440; doi:10.1126/sciadv.adk6583)
Supplement: Supplementary file 1 — Supplementary Discussion Figs. S1 to S7 Tables S2 and S3 Legend for table S1 Legends for movies S1 to S9 References [file sciadv.adk6583_sm.pdf]

Science Advances
AAAS

Supplementary Materials for

TNIK: A redox sensor in endothelial cell permeability

Justin Joachim *et al.*

Corresponding author: Aleksandar Ivetic, aleksandar.ivetic@kcl.ac.uk

Sci. Adv. **10**, eadk6583 (2024)
DOI: 10.1126/sciadv.adk6583

The PDF file includes:

Supplementary Discussion
Figs. S1 to S7
Tables S2 and S3
Legend for table S1
Legends for movies S1 to S9
References

Other Supplementary Material for this manuscript includes the following:

Table S1
Movies S1 to S9

Supplemental Discussion

Activation loop exchange and trans-autophosphorylation as a model for TNIK activation

Kinases must be activated before they can phosphorylate their substrates and this process commonly occurs through phosphorylation of the activation loop (A-loop) of the kinase, which stabilises the A-loop in a conformation that allows kinase-substrate binding (*100*). Indeed, this is likely the case for TNIK as mutations of the A-loop phosphorylation sites T181A or T187A inhibit TNIK kinase activity towards its substrate (*39*). The positions of the phosphorylated A-loop residues T181 and T187 in the active TNIK kinase domain (PDB: 6RA7) are shown in **movie S6**. The TNIK structure contains a symmetry-related dimer where the C-terminal section of the A-loops of each monomer are contacting one another (**fig. S7A-B**). Through POPSCOMP (*94*) and PISA (*56*) analysis, and similarly to what has been published for the structure of the TNIK homologue MAP4K4 (*101*, *102*), four A-loop residues (187-TFIGT-191) were identified in monomer A that are packing against the same residues in monomer B in an anti-parallel fashion, forming a series of hydrogen bonds (**fig. S7A-B**). Each Thr187 main chain carbonyl interacts with Thr191-OG1 from the other monomer; additionally, each Ile-189 main chain nitrogen, hydrogen bonds to Gly190 main chain carbonyl from the neighbouring molecule (**fig. S7B**).

The A-loop conformation of the monomers suggests that the TNIK kinase domain can dimerise in a 'face-to-face' fashion. A mechanism where kinase domain A-loops are exchanged in a 'face-to-face' homodimer and autophosphorylation occurs in trans, has been described for CHK2, SLK, LOK, MST4 and DAPK3 (*100*, *103*, *104*). Some of these kinases, like TNIK, also phosphorylate ERM proteins (*105-107*). During this trans-activation mechanism (*100*), which we propose for TNIK, dimerisation of the inactive kinase promotes A-loop exchange between monomers, producing an active conformation in both kinase molecules in trans; trans-autophosphorylation of the exchanged A-loops occurs resulting in an activated dimeric kinase (**fig. S7C**). Once reciprocally phosphorylated, the kinase continues to target downstream substrates. During dimeric trans-autophosphorylation, the inactive and partially disordered A-loop of one monomer reaches into the substrate binding site of the other kinase monomer (**fig. S7C**). In this manner, it is likely that TNIK Thr181 and Thr187 from one kinase A-loop can be positioned at the catalytic site of the second kinase molecule to be phosphorylated. Whilst the crystal structure of TNIK (PDB: 5CWZ) supports the A-loop exchange autophosphorylation model (*100*), it probably represents an

intermediate pre-catalytic state, similarly to what has been described for a structure of the MST4 dimer (*104*). Activation and catalysis require correct positioning of the αC-helix and establishment of the salt-bridge between K54 and E69 (in TNIK), which are not present here.

Kinases that are activated by the A-loop exchange mechanism are thought to be homodimeric in their active states (*100*). Indeed, SLK, LOK and DAPK3 contain coiled-coil domains, C-terminal to their kinase domains, that promote constitutive dimerisation (*100*). Similarly, TNIK contains a coiled-coil domain C-terminal to its kinase domain, and deletion of the N-terminal kinase domain alone does not prevent TNIK homodimerisation (*57*, *108*). It remains to be experimentally determined whether the coiled-coil domain of TNIK promotes dimerisation and kinase activation, and how TNIK homodimerisation may be regulated (e.g. by signalling and scaffolding). Indeed, experimentally-induced homodimerisation of SLK promotes its kinase activity and mutation of SLK to its monomeric form inhibits autophosphorylation ability (*105*, *109*). Nevertheless, the TNIK kinase domain homodimer (PDB: 5CWZ) is predicted to be stable by both POPSCOMP (*94*) and PISA (*56*) analysis; the interaction interface by PISA is scored with the maximum 1.000 complex formation significance score, predicting that the dimer interface plays an essential role in complex formation. A large accessible surface area that is predominantly hydrophobic, ranging from 1441.8 $Å^2$ for monomer A to 1415.6 $Å^2$ for monomer B, is buried in each of the dimer interfaces; this is in the range typically found for functional protein-protein interactions.

The A-loop exchange model of activation is driven by homodimerisation and therefore does not require A-loop phosphorylation sites to adhere to the kinase's substrate consensus sequence. TNIK recognises and phosphorylates the substrate consensus sequences pT/S-L/I/V-D/E-x-x-x-R/K, pT/S-L/I/V-x-R/K and pT/S-L-P/Q-L/I-x-x-R/K (*39*). In concordance with the A-loop exchange model, TNIK A-loop residue Thr187 (187-TFIGT-191) does not conform to the TNIK substrate consensus sequence.

A model for TNIK kinase inactivation by cysteine oxidation and disulphide formation

Experimental biochemical data and molecular dynamics (MD) simulations suggest that in the TNIK dimer, apposed C202 residues come in close enough proximity to allow reversible disulphide bond formation (**fig. 5-6**, **fig. S3** and **movies S3&S4**). However, in this MD simulated structure the A-loop swapped hydrogen bond network cannot form (compare **fig. S7D-E** with **fig. S7A-B**). Instead,

conformational changes favour the formation of a disulphide bridge between C202-C202 residues (3.5 Å distance). Conformational changes induced by kinase-substrate interactions at remote sites are required for adoption of the active kinase conformation; this is prevented in TNIK containing a C202-C202 disulphide bond. Furthermore, the activating phosphorylation sites of the A-loop are trapped in the dimer interface, where they are inaccessible for regulation by kinases or phosphatases (**fig. S7E**). In the intermediate pre-catalytic state (PDB: 5CWZ), stabilisation of this A-loop hydrogen bond network prevents the association of C202-C202 residues (11.8 Å apart) (**fig. S7A-B**).

We propose a model where oxidised and inactive disulphide linked TNIK is in equilibrium with activated TNIK via an intermediate pre-catalytic transition state (**fig. S7F**). TNIK disulphide bond formation prevents the formation of the intermediate pre-catalytic state and A-loop exchange. TNIK reduction allows formation of the intermediate pre-catalytic state, followed by A-loop exchange and trans-autophosphorylation. This leads to unmasking of the dimerisation interface and adoption of the fully active TNIK conformation (PDB: 5AX9), where additional kinase-kinase and kinase-substrate signalling continues.

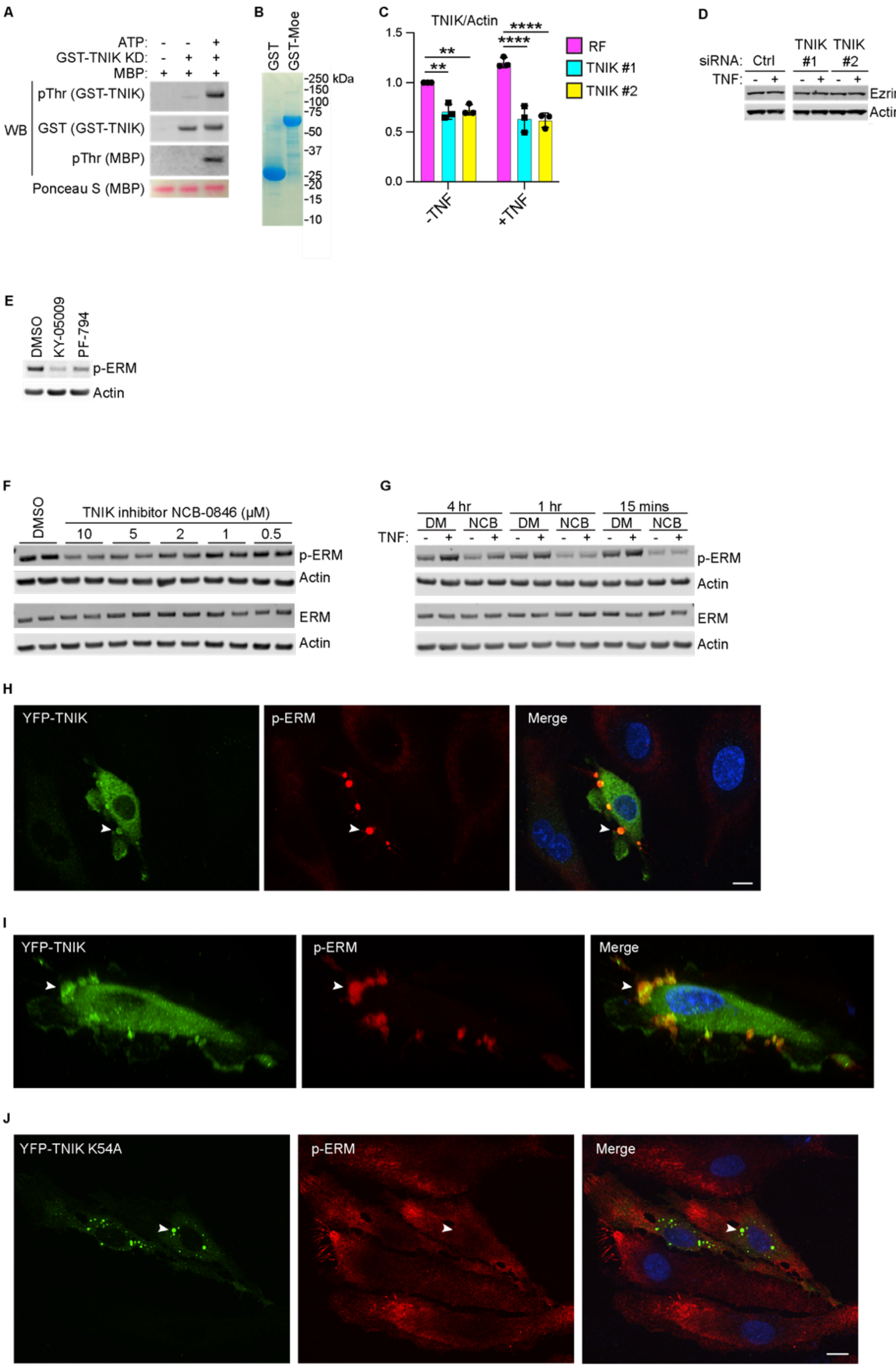

Figure S1) TNIK mediates ERM activation in endothelial cells. Related to **fig. 1**.

A GST-TNIK kinase domain mixed with myelin basic protein (MBP) and subjected to in vitro kinase assays before Western blotting. KD = kinase domain, pThr = phospho-threonine, MBP = myelin basic protein (a known TNIK substrate). Ponceau S allows visualisation of sample loading. **B** GST or GST-Moesin C-terminal domain expressed and purified from E. coli and subjected to SDS-PAGE and Coomassie staining. **C** HUVEC transfected with control RISC-free (RF) siRNA or siRNA duplexes targeting TNIK and stimulated with TNF-α for 15 min prior to Western blotting. Quantification of Western blot of TNIK siRNA in **fig. 1F**, two-way ANOVA, mean ± SEM, **** $P \leq 0.0001$, $n = 3$ independent experiments. **D** HUVEC transfected with siRNA duplexes to TNIK and stimulated with TNF-α for 15 min prior to Western blotting. **E** HUVEC were treated with DMSO vehicle control or the TNIK inhibitors KY-05009 or PF-794 for 1 hr before Western blotting. **F** HUVEC were treated with DMSO vehicle control or the indicated concentration of TNIK inhibitor NCB-0846 and 20 ng/ml TNF-α for 15 min before Western blotting. **G** HUVEC were treated with DMSO vehicle control (DM) or 10 μM TNIK inhibitor NCB-0846 (NCB) and 20 ng/ml TNF-α for the indicated times before Western blotting. **H** HUVEC expressing YFP-TNIK were fixed and labelled with anti-p-ERM or anti-GFP (for detection of YFP-TNIK) antibodies prior to confocal microscopy. Arrowhead, YFP-TNIK and p-ERM colocalisation at endothelial cell plasma membrane bleb. Scale bar, 10 μm. **I** HUVEC expressing YFP-TNIK were fixed and labelled with anti-p-ERM or anti-GFP (for detection of YFP-TNIK) antibodies prior to confocal microscopy. Arrowhead, YFP-TNIK and p-ERM colocalisation at endothelial cell plasma membrane bleb. A 3D volumetric rendering of a Z-stack in isometric view is displayed. J HUVEC expressing YFP-TNIK K54A kinase-dead mutant were fixed and labelled with anti-p-ERM or anti-GFP (for detection of YFP-TNIK) antibodies prior to confocal microscopy. Arrowhead, cytoplasmic YFP-TNIK K54A punctum devoid of p-ERM staining. Scale bar, 10 μm.

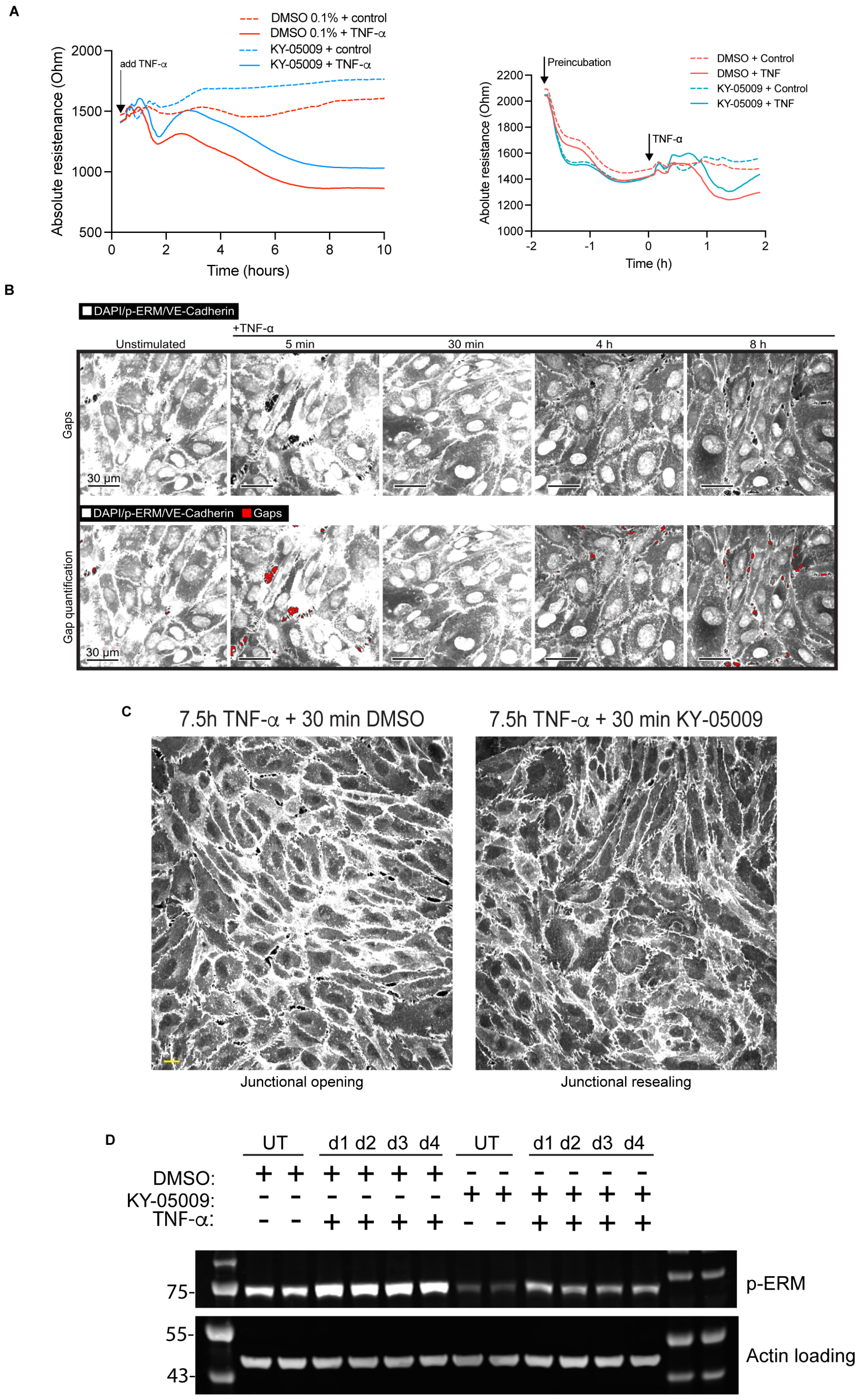

Figure S2) TNIK regulates permeability and paracellular gap formation in HUVEC. Related to **fig. 2D** and **fig. 3A-E**.

A Left-hand trace - absolute endothelial resistance values of HUVEC pre-treated with 10µM KY-05009 (2 h) followed by TNF-α stimulation (10 ng/mL, arrow). Mean and 95% confidence interval of 12 biological repeats from n=4 independent experiments. Right-hand trace, same as the left-hand trace, but brought back for 2h before the addition of TNF-α to show stable traces 45 min before addition of TNF-α. **B** HUVEC monolayers were stimulated with 10 ng/ml of TNF-α for indicated time points and confocal microscopy enabled the visualisation of cell nuclei (DAPI), p-ERM and VE-cadherin. See methods on details of how volume of gaps were acquired. Upper panel shows presence of paracellular gaps in black after converting DAPI, VE-cadherin and p-ERM signals to white. Lower panel of images show where gaps have been detected and used for analysing gap volume as a percentage of the total cell area. **C** HUVEC monolayers were first treated with 7.5 h TNF-α then with 30 min incubation with either DMSO carrier or with 10 µM KY-05009. Cells were subsequently fixed and imaged for VE-cadherin and converted into black and white images to monitor paracellular gaps. Left and right images represent a wider field of view of **fig. 3B**. Yellow scale bar = 15 µm. **D** Western blot of HUVEC monolayers treated as in **C** (i.e. TNF-α first for 7.5 h, followed by 30 min incubation with KY-05009). Quantification of p-ERM levels between the DMSO and KY-05009-treated groups is shown in **fig. 3C**. Four independent experiments performed on 4 different days (d = day). Basal p-ERM levels are significantly reduced under conditions where KY-05009 was incubated for 30 min but TNF-α was not added (UT = untreated – media added without TNF-α). The last two lanes in **D** correspond to molecular weight markers.

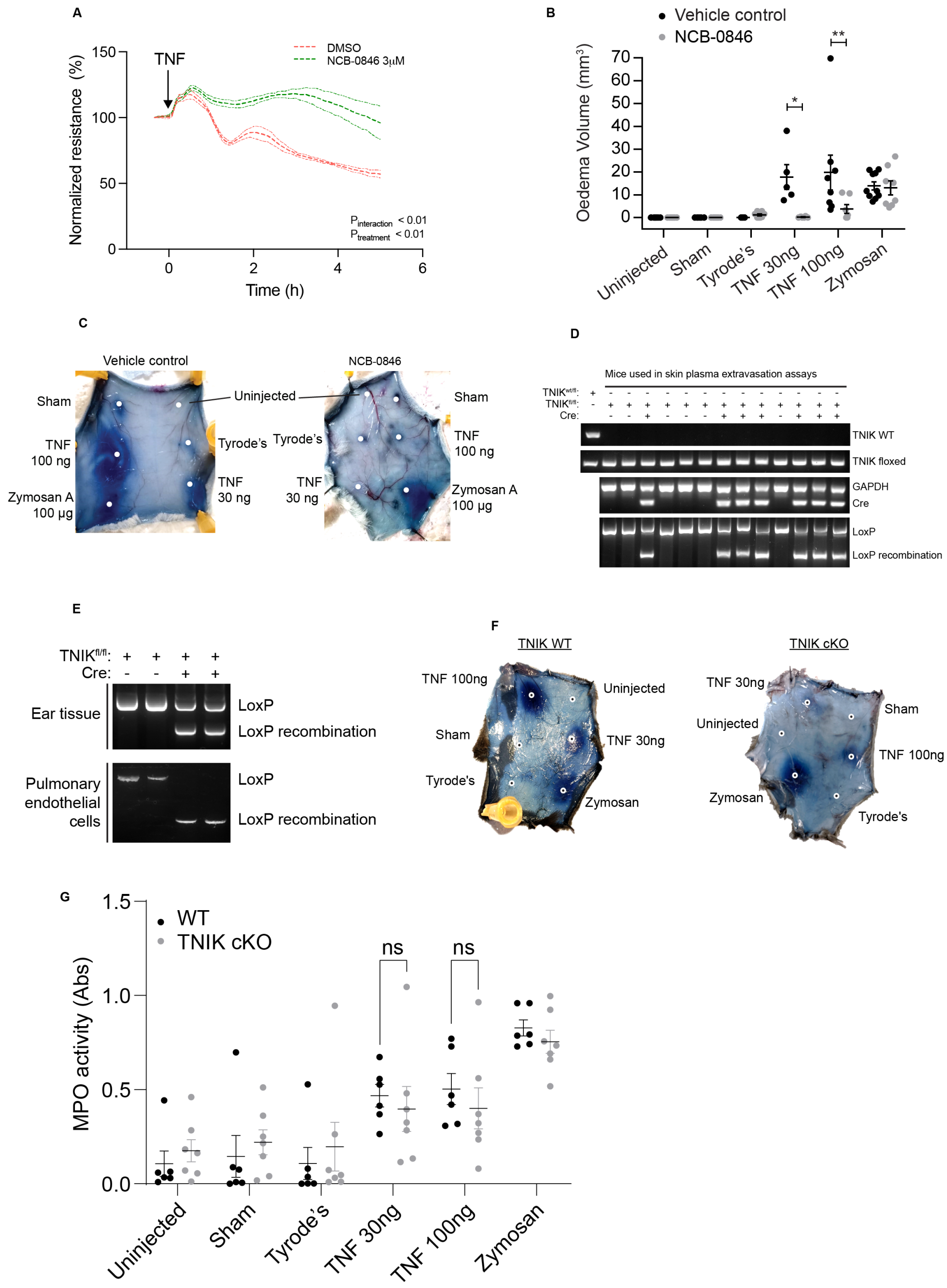

Figure. S3) Pharmacologic or genetic inhibition of TNIK suppresses TNF-α-induced permeability. Related to **fig. 3 F&G**.

A Endothelial Cell-substrate Impedance Sensing (ECIS) traces, demonstrating that NCB-0846 inhibits TNF-α-induced permeability in HUVEC monolayers. HUVEC monolayers were subjected to TNIK inhibition by NCB-0846 for 2h prior to challenge with TNF-α. NCB-0846 has been shown to be used in vivo at lower doses than KY-05009 (*38*). Data represent the mean of 12 biological repeats from n=4

independent experiments. **B** CD1 mice were treated with DMSO vehicle control or TNIK inhibitor NCB-0846 by intraperitoneal (IP) injection followed by TNF-α or zymosan-induced skin permeability assay as outlined in Methods section and (*40*). n = 5-10 CD1 mice per group. Statistical analysis using two-way ANOVA, mean ± SEM, * $P \leq 0.05$, ** $P \leq 0.01$. **C** Representative qualitative assessment of plasma extravasation by Evans Blue dye leakage in the dorsal skin from CD1 mice used in **B**. Injection points are indicated with white dots. **D** Ear biopsy genotyping confirmation of mice used in **fig. 3G**. **E** Genotyping of isolated pulmonary microvascular endothelial cells (isolated using cell isolation kit (see Methods and **tables S2 and S3** for primer sequences for genotyping)) to confirm complete endothelial-specific knockout of endogenous TNIK in mice used in **fig. 3G**. **F** Representative qualitative assessment of plasma extravasation by Evans Blue dye leakage in the dorsal skin from tamoxifen-inducible conditional knockout mice used in **fig. 3G**. **G** Assessment of neutrophil-derived myeloperoxidase (MPO) activity derived from skin biopsy punches isolated from mice used in **fig. 3G** (see Methods section and (*40*) for more information). n = 6-7 mice per group. Statistical analysis using two-way ANOVA, ns = non-significant.

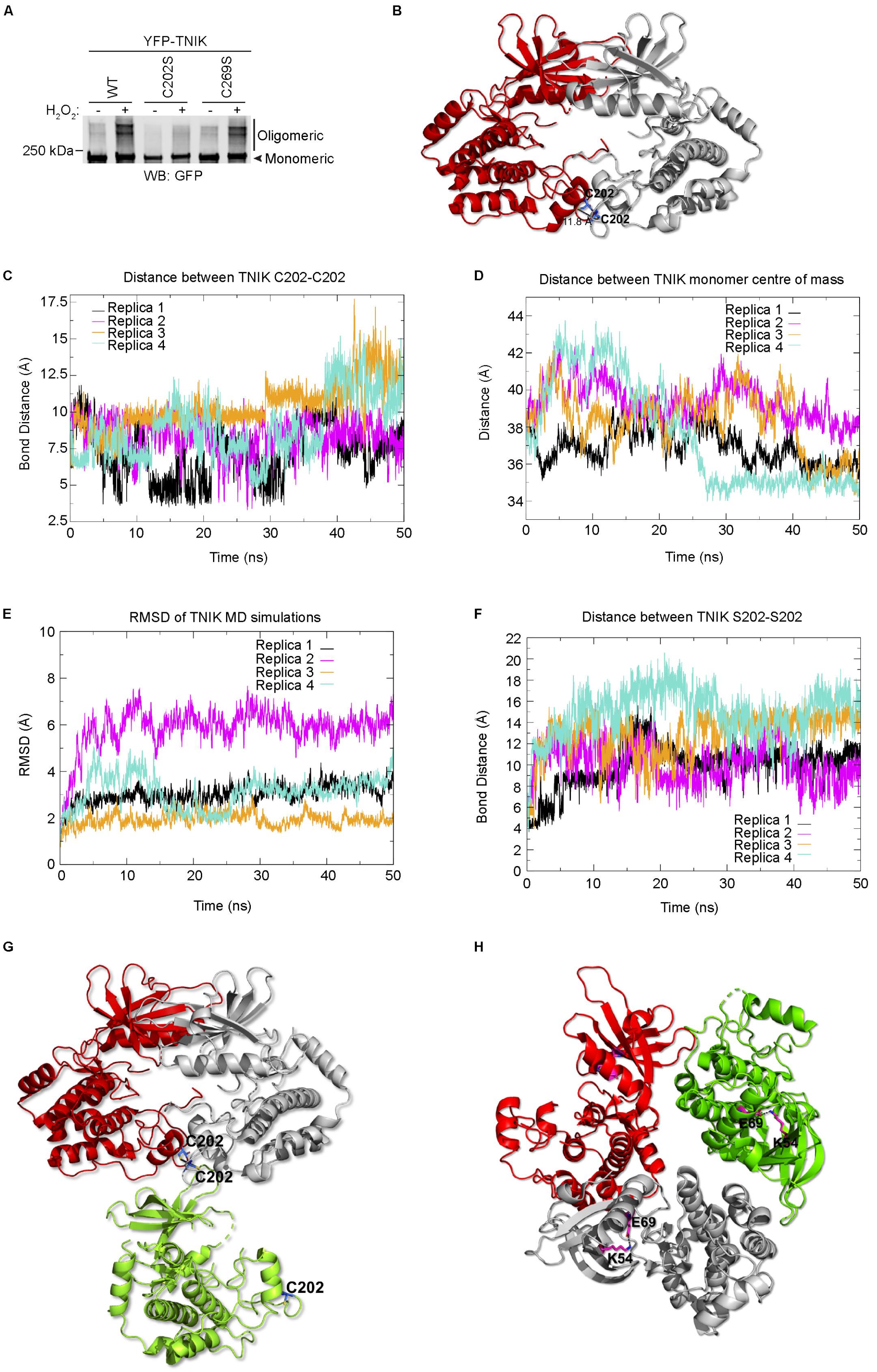

Figure. S4) Molecular dynamics simulations predict TNIK C202 residues can form reversible disulphide bonds. Related to **fig. 6**.

A YFP-TNIK, or YFP-TNIK mutants, were expressed in HEK293T cells by plasmid transfection and immunoprecipitated. Immunoprecipitates were oxidised with 200 µM hydrogen peroxide at 30°C for 15 min followed by non-reducing SDS-PAGE and Western blotting. **B** Crystal structure containing TNIK dimer (PDB: 5CWZ), face-to-face monomers shown in different colours with positions of adjacent Cys202 residues on loops. Chain C from the crystal is omitted. **C** Distance between apposed C202 residue sulphur atoms in docked wt-TNIK dimer during 50 ns molecular dynamics simulations. Four replica simulations are depicted. **D** Time evolution of the distance between the centres of mass of chain A and chain B in the TNIK dimer, relating to molecular dynamics simulations in **C**. **E** The root mean square deviation (RMSD) of C-alpha atoms of the dimer backbone was computed with respect to the first frame docked structure in each molecular dynamics simulation from **C**. Least squares fitting of the C-alpha atoms as a docked dimer was performed. The RMSD depicts the TNIK dimer conformation variation along the trajectory, in comparison to the first frame docked structure. **F** Distance between apposed in silico mutated S202 residues (side chain hydroxyl hydrogen atoms) in mt-TNIK docked dimer, during 50 ns molecular dynamics simulations. Four replica simulations are depicted. **G** Inactive TNIK kinase domain crystal structure (PDB: 5CWZ) showing positions of C202 in each chain (differently coloured). Polypeptide chains in TNIK dimer are shown in red and grey. Chain C from the crystal is shown in green. **H** Active TNIK kinase domain crystal structure (PDB: 5AX9) showing activating salt bridge between K54 and E69.

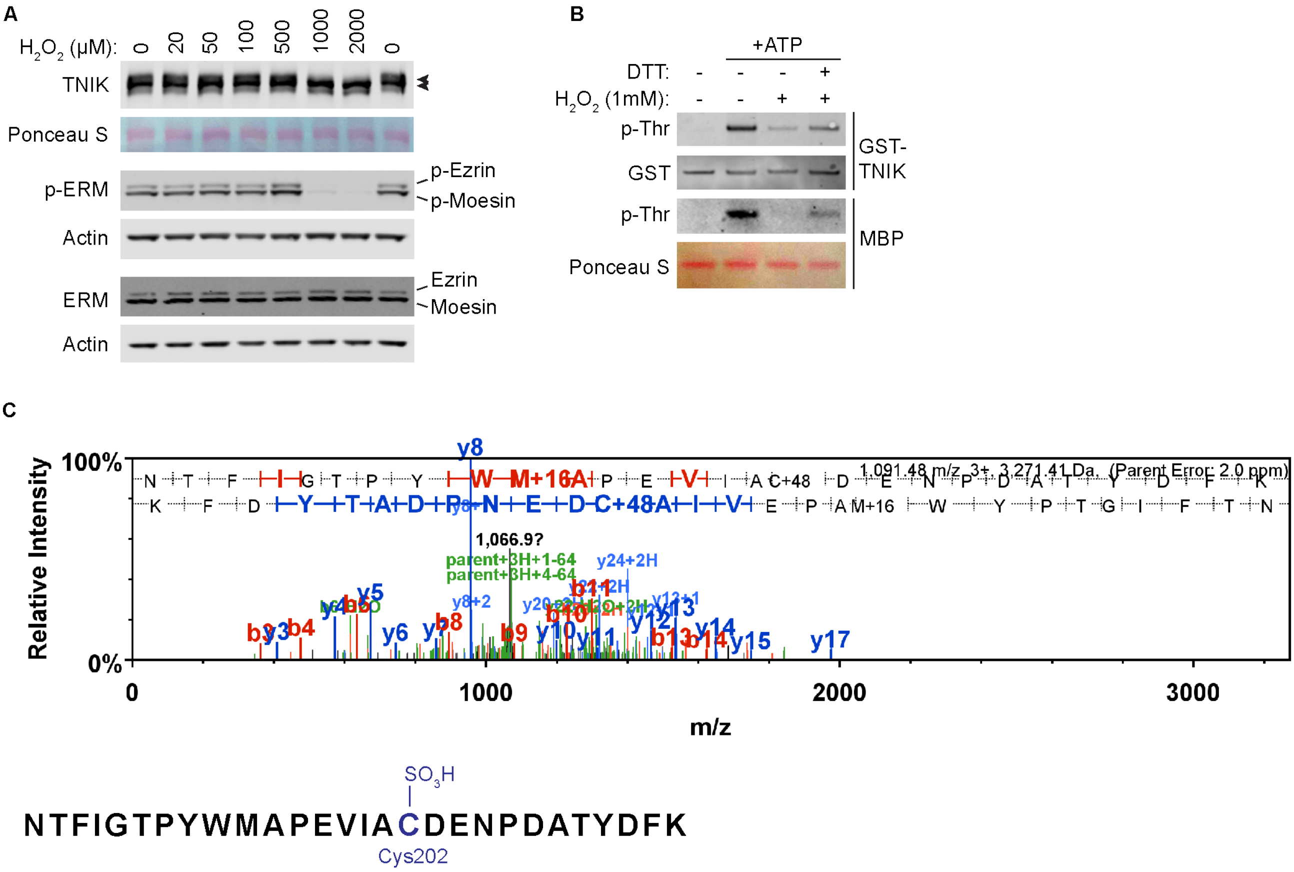

Figure. S5) High concentrations of hydrogen peroxide irreversibly deactivate TNIK activity and terminally oxidise TNIK C202. Related to **fig. 7**.

A HUVEC monolayers were treated with the indicated concentration of hydrogen peroxide for 15 min followed by SDS-PAGE and Western blotting. Arrowheads, differentially migrating species of TNIK by SDS-PAGE. **B** GST-TNIK kinase domain was oxidised with the indicated concentration of hydrogen peroxide, followed by reduction with DTT where indicated. In vitro kinase assays were then performed, followed by Western blotting. **C** Mass spectrometry identification of terminally oxidised TNIK C202 (Cys202) to sulphonic acid (trioxidation – SO_3H). Parent ion error 2 ppm. A strongly matched set of blue peaks which are assigned to the y-ions from the C-terminal end of the peptide in a sequence string from y4-y15 are observed, including the correctly assigned modification at y12 of the additional 48 Da (trioxidation) on TNIK C202.

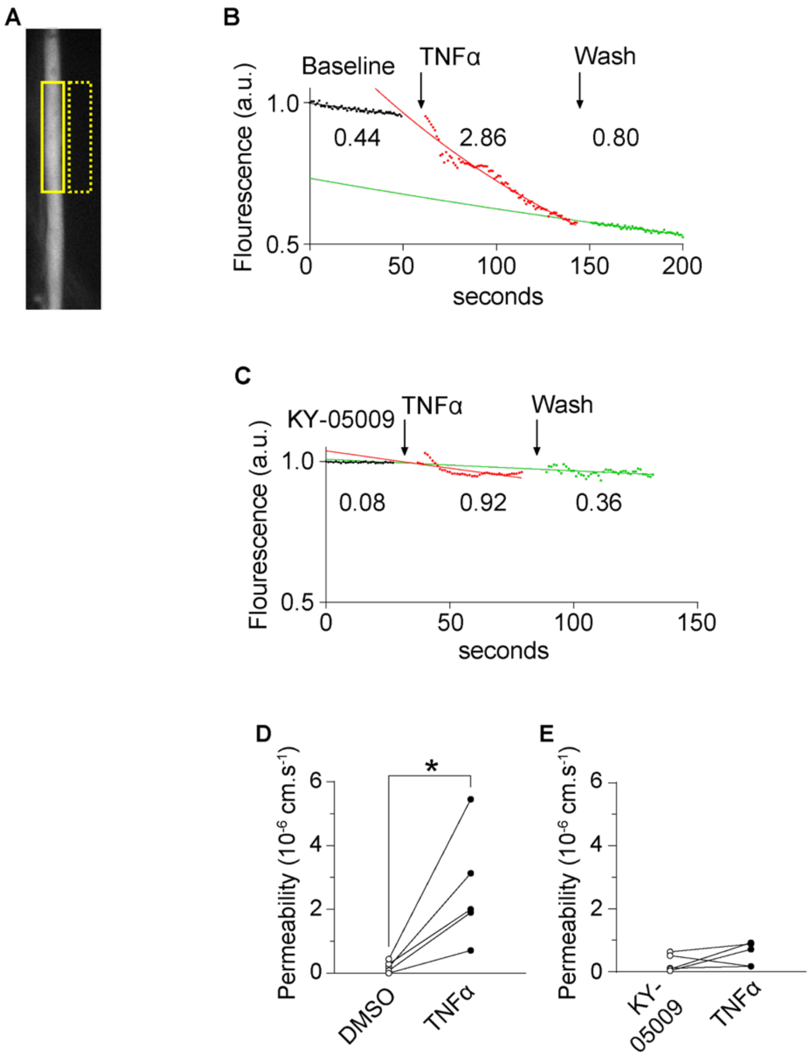

Figure. S6) A cadaver model used to explore permeability of the cremasteric microcircularion. Related to the Discussion section.

A The isolated cremaster muscle microvasculature was loaded with FITC-albumin and allowed to stabilise. A series of images were captured, showing one venule (20µm diameter) just as TNF-α application commenced. The gradient of FITC-albumin across the vessel wall was obtained from the difference in mean pixel values within the yellow rectangles, over the vessel (solid yellow line) and the adjacent interstitium (dashed yellow line) and was normalised to the value of 1. **B** Kinetic profile of microvessel permeability from a single experiment. The baseline was obtained from superfusion of buffer containing DMSO (carrier control). TNF-α was applied between the indicated arrows, which resulted in dye loss from the vessel lumen within seconds. This loss ceased soon after TNFα was washed-out (indicated as "wash" in the figure) by superfusion of the cremaster in buffer. Single phase exponentials were fitted to obtain the

rate constants, from which the permeability was calculated (values are shown adjacent to the curves). **C** Buffer containing KY-05009 was superfused for 10 minutes before TNF-α was applied. **D-E** Data are expressed as the sum of 5 independent vessels that were analysed – each derived from an individual mouse. * = $p<0.05$ (paired student ‘t’ test).

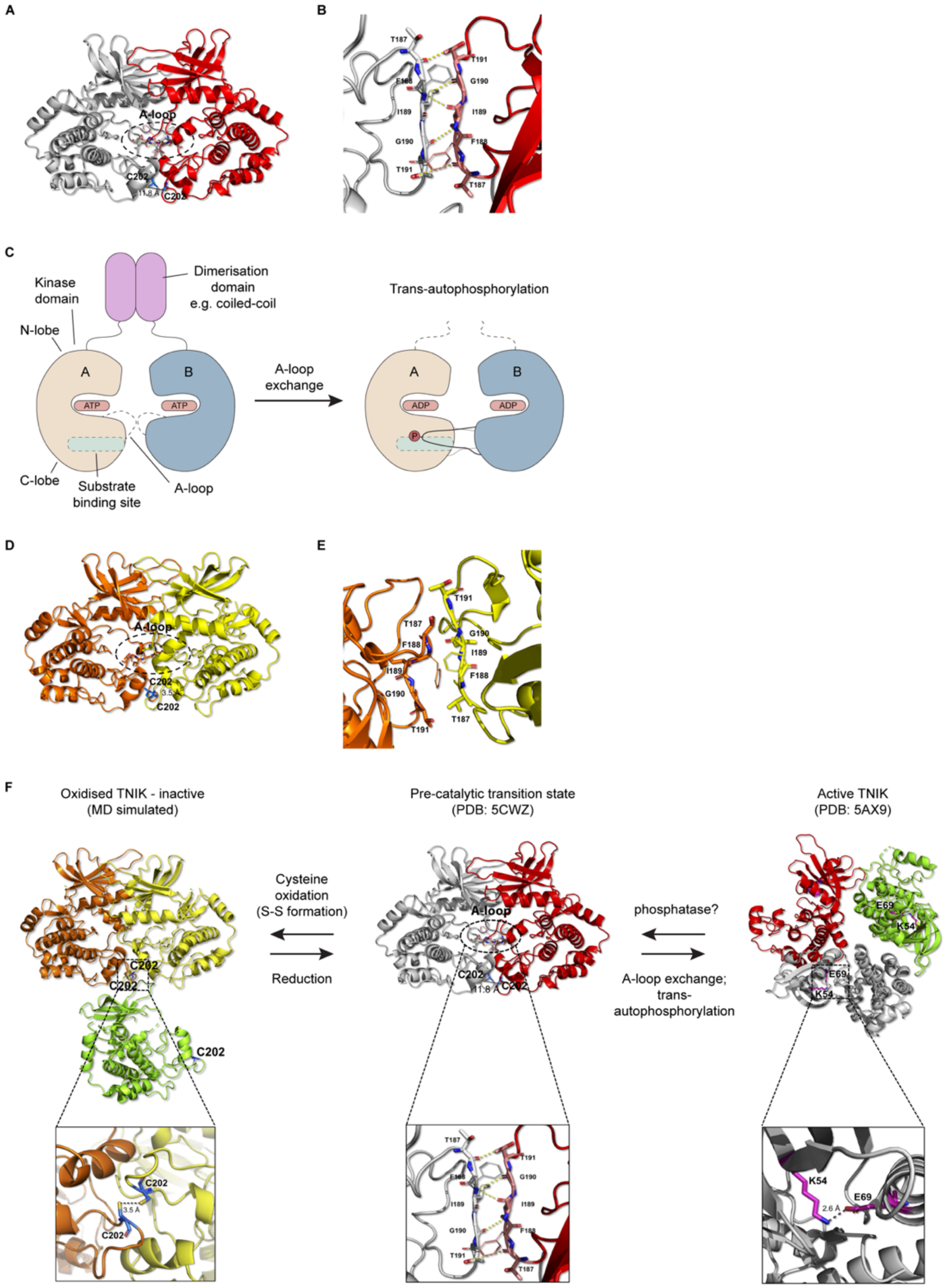

Figure. S7) Activation loop exchange and cysteine oxidation as a model for TNIK activation and inactivation, respectively. Related to Supplemental Discussion.

A TNIK crystal structure (PDB: 5CWZ) dimerisation. Chain A is shown in grey, chain B is shown in red, overall packing. A-loop, activation loop. Distance between juxtaposed C202 sulphur atoms is shown. Chain C from crystal is omitted. **B** Molecular interactions seen as the interface between antiparallel A-loops, face-to-face dimer, related to A. **C** TNIK dimerisation, which in addition to the kinase domain may also be aided by the TNIK coiled-coil domain, promotes activation loop exchange between adjacent kinase domains, producing an active confirmation in both kinase molecules in trans. Phosphorylation of the exchanged activation loops is then able to occur through trans-autophosphorylation. Based on model from Oliver et al. (*100*) **D** Molecular dynamics simulation of TNIK dimer showing apposed C202 residues within the threshold distance (3.5 Å minimum reached) to allow reversible disulphide bond formation. A-loop, activation loop. **E** Disrupted hydrogen bonding seen between the antiparallel A-loops, face-to-face dimer, related to D. **F** Proposed model for TNIK inactivation driven by cysteine oxidation (disulphide formation) and activation driven by dimeric A-loop exchange. Left, molecular dynamics simulation of TNIK dimer showing apposed C202 residues within the threshold distance (3.5 Å minimum reached) to allow reversible disulphide bond formation. Chain C (green) of the inactive crystal structure (PDB: 5CWZ) is superimposed. MD, molecular dynamics. Middle, crystal structure of inactive TNIK kinase domain dimer (PDB: 5CWZ) showing A-loop contacts prior to trans-autophosphorylation. Right, crystal structure of active TNIK kinase domain (PDB: 5AX9) showing activating K54-E69 salt bridge.

Table S1) Kinome-wide RNAi screen in Drosophila melanogaster S2R$^+$ cells using p-Dmoesin staining as a readout. Related to **fig. 1**.

Primer Name	Primer Sequence
Forward primer: TNIK LoxP_F	TCAGGTCCCCACAGTAAAGG
Reverse primer: TNIK LoxP_R	TGTCAGGGTGAAATGGGGTA

Table S2) Sequences of primers used to assess the efficiency of TNIK knockout

Amplification Reaction	Primer Name	Primer Sequence
**TNIK WT allele**	Forward primer: TNIK-49289_F	TCAGGTCCCCACAGTAAAGG
	Reverse primer: TNIK-49289_R	AACCTGGCTTCACCAAGTGT
**TNIK Floxed allele**	Forward primer: TNIK-49289_F	TCAGGTCCCCACAGTAAAGG
	Reverse primer: CAS_R1_Term	TCGTGGTATCGTTATGCGCC
**Cre transgene/GAPDH**	Cre-Forward primer	TGCCAGGATCAGGGTTAAAG
	Cre-Reverse primer	CCCGGCAAAACAGGTAGTTA
	GAPDH-Forward primer	CCTAGACAAAATGGTGAAGG
	GAPDH-Reverse primer	GACTCCACGACATACTCAGC

Table S3) Sequences of primers used for TNIK genotyping as recommended by the Sanger Institute

Movie S1) Polarised distribution of constitutively activated moesin-GFP expressed in HUVEC. Related to **fig. 4C**.

Spinning disk confocal live cell imaging of HUVEC lentivirally transduced with TD Moesin-GFP to determine the subcellular distribution of this construct in endothelial cells. The polarised distribution of TD moesin-GFP is only detected in migrating HUVEC. Monolayers were imaged for a period of 10 hr and 6 min. Scale bar = 66 μm.

Movie S2) H_2O_2 induces YFP-TNIK relocalisation to cytoplasmic foci. Related to **fig. S1J and fig. 5E**.

Widefield live cell imaging of HUVEC transfected with YFP-TNIK. Cells on left: HUVEC were imaged for 30 mins prior to the addition of H_2O_2. Cells on right: the same cells were imaged for 1 hr, immediately after the addition of 100 μM H_2O_2.

Movie S3) Comparison of apposed C202 resides in TNIK dimer between the inactive TNIK crystal structure and molecular dynamics simulation of the TNIK docked model. Related to **fig. 6A-C**, **fig. S4B**, and **movie S4**.

Crystal structure of the dimeric inactive TNIK kinase domain (PDB: 5CWZ) is shown with monomers coloured grey and red and apposed C202 sulphur atom distance is 11.8 Å. This is superimposed onto the docked model of the TNIK structure after molecular dynamics simulation with monomers coloured yellow and orange and apposed C202 residues reach a minimum distance of 3.5 Å (see also **fig. 6A-B**). RMSD of superimposed structures is 3.9 Å indicating no significant global conformational changes between the two structures. H-bonding between contacting A-loop residues at the dimer interface is shown.

Movie S4) Molecular dynamics simulation of the wt-TNIK docked model showing apposed C202 residues in the TNIK kinase domain dimer. Related to **fig. 6** and **movie S3**.

The docked model of the dimeric inactive TNIK kinase domain structure during molecular dynamics simulation with monomers coloured yellow and orange. Apposed C202 residues are depicted as blue sticks

with distance between cysteine sulphur atoms displayed, reaching a minimum distance of 3.5 Å. Left, whole dimer view; right, magnified view. Movie relates to **fig. 6A-B**. Frames shown in the movie were taken every 100 ps from the simulation.

Movie S5) Molecular dynamics simulation of the mt-TNIK docked model showing apposed in silico mutated S202 residues in the TNIK kinase domain dimer. Related to **fig. 6**.

The docked model of the dimeric inactive TNIK kinase domain structure during molecular dynamics simulation with monomers coloured yellow and orange. C202S mutations were introduced into both monomers. S202 residues as red sticks, with the distance between hydroxyl hydrogen atoms displayed. Left, whole dimer view; right, magnified view. Movie relates to **fig. 6D-E**. Frames shown in the movie were taken every 100 ps from the simulation.

Movie S6) General structural features of kinases shown with the active TNIK kinase domain (PDB: 6RA7).

TNIK follows the canonical kinase domain architecture which encompasses two subdomains: the N-terminal lobe composed of mostly β-sheets and the C-terminal lobe comprised predominantly of α-helices, which are connected by a flexible linker (hinge). The N-lobe contains only one α-helix, the αC helix, of which position has a pivotal regulatory role in the kinase. The αC helix rotates inwards towards the ATP-binding pocket (αC-in conformation) to facilitate the formation of a conserved salt-bridge present among kinases between a lysine in the β3 strand (K54 in TNIK) and a glutamate in the αC helix (E69 in TNIK). The formation of this salt-bridge is key for enzyme activity as it allows substrate access and ATP binding. The αC-in conformation is a structural feature present in active kinase domains and this signature is used to determine their activation state. Another structural signature is the activation loop (A-loop) where in an active state its conformation is extended away from the kinase domain to allow substrate binding at the substrate binding site. The phosphate-loop (p-loop) is a conserved sequence motif that contributes to the binding of nucleotides. In TNIK, threonines at 181 and at 187 are autophosphorylation sites that stabilise the A-loop's active conformation.

Movie S7) Inhibition of endogenous ROS in HUVEC monolayers promotes endothelial cell retraction, blebbing and excessive paracellular gap formation. Related to **fig. 8** and **movie S8**.

Brightfield live cell imaging of a HUVEC monolayer treated with 10 μM of the pan-flavoenzyme inhibitor, diphenyleneiodonium chloride (DPI). Movie starts after the addition of DPI and lasts 1 hr. Inset shows endothelial cell undergoing retraction and extensive plasma membrane blebbing.

Movie S8) TNIK inhibition reverses the effect of DPI and promotes endothelial cell membrane extension, inhibition of blebbing and reduced paracellular gaps. Related to **fig. 8** and **movie S7**.

This movie follows on from **movie S7**. Brightfield live cell imaging of a HUVEC monolayer treated with 10 μM DPI, followed by treatment with 10 μM KY-05009. Movie starts after the addition of KY-05009 and lasts 2 hr 20 min. Inset shows endothelial cell undergoing extensive plasma membrane blebbing, which is reversed after the addition of TNIK inhibitor.

Movie S9) Simultaneous inhibition of TNIK using 10 μM KY-05009 and endogenous ROS production with 10 μM DPI, prevents endothelial cell membrane retraction, blebbing and formation of large paracellular gaps. These videos demonstrate that adding DPI to HUVEC is not causing cell rounding due to drug-induced toxicity. Brightfield live cell imaging of three HUVEC monolayers, starting after addition of the indicated treatments: DPI alone or simultaneously with the TNIK inhibitors KY-05009 or PF-794. Movies are looped 3 times and each loop lasts 1 hr.

REFERENCES AND NOTES

1. L. Claesson-Welsh, Vascular permeability--the essentials. *Ups. J. Med. Sci.* **120**, 135–143 (2015).

2. Y. Komarova, A. B. Malik, Regulation of endothelial permeability via paracellular and transcellular transport pathways. *Annu. Rev. Physiol.* **72**, 463–493 (2010).

3. Y. A. Komarova, K. Kruse, D. Mehta, A. B. Malik, Protein interactions at endothelial junctions and signaling mechanisms regulating endothelial permeability. *Circ. Res.* **120**, 179–206 (2017).

4. S. Sukriti, M. Tauseef, P. Yazbeck, D. Mehta, Mechanisms regulating endothelial permeability. *Pulm. Circ.* **4**, 535–551 (2014).

5. M. Kellner, S. Noonepalle, Q. Lu, A. Srivastava, E. Zemskov, S. M. Black, ROS signaling in the pathogenesis of acute lung injury (ALI) and acute respiratory distress syndrome (ARDS). *Adv. Exp. Med. Biol.* **967**, 105–137 (2017).

6. B. T. Thompson, R. C. Chambers, K. D. Liu, Acute respiratory distress syndrome. *N. Engl. J. Med.* **377**, 1904–1905 (2017).

7. C. Park-Windhol, P. A. D'Amore, Disorders of vascular permeability. *Annu. Rev. Pathol.* **11**, 251–281 (2016).

8. R. G. Fehon, A. I. McClatchey, A. Bretscher, Organizing the cell cortex: The role of ERM proteins. *Nat. Rev. Mol. Cell Biol.* **11**, 276–287 (2010).

9. A. I. McClatchey, ERM proteins at a glance. *J. Cell Sci.* **127** (Pt. 15), 3199–3204 (2014).

10. M. Koss, G. R. Pfeiffer, Y. Wang, S. T. Thomas, M. Yerukhimovich, W. A. Gaarde, C. M. Doerschuk, Q. Wang, Ezrin/radixin/moesin proteins are phosphorylated by TNF-α and modulate permeability increases in human pulmonary microvascular endothelial cells. *J. Immunol.* **176**, 1218–1227 (2006).

11. M. Magendantz, M. D. Henry, A. Lander, F. Solomon, Interdomain interactions of radixin in vitro. *J. Biol. Chem.* **270**, 25324–25327 (1995).

12. R. Gary, A. Bretscher, Ezrin self-association involves binding of an N-terminal domain to a normally masked C-terminal domain that includes the F-actin binding site. *Mol. Biol. Cell* **6**, 1061–1075 (1995).

13. M. A. Pearson, D. Reczek, A. Bretscher, P. A. Karplus, Structure of the ERM protein moesin reveals the FERM domain fold masked by an extended actin binding tail domain. *Cell* **101**, 259–270 (2000).

14. B. T. Fievet, A. Gautreau, C. Roy, L. Del Maestro, P. Mangeat, D. Louvard, M. Arpin, Phosphoinositide binding and phosphorylation act sequentially in the activation mechanism of ezrin. *J. Cell Biol.* **164**, 653–659 (2004).

15. C. Barret, C. Roy, P. Montcourrier, P. Mangeat, V. Niggli, Mutagenesis of the phosphatidylinositol 4,5-bisphosphate (PIP(2)) binding site in the NH(2)-terminal domain of ezrin correlates with its altered cellular distribution. *J. Cell Biol.* **151**, 1067–1080 (2000).

16. M. Algrain, O. Turunen, A. Vaheri, D. Louvard, M. Arpin, Ezrin contains cytoskeleton and membrane binding domains accounting for its proposed role as a membrane-cytoskeletal linker. *J. Cell Biol.* **120**, 129–139 (1993).

17. F. Nakamura, L. Huang, K. Pestonjamasp, E. J. Luna, H. Furthmayr, Regulation of F-actin binding to platelet moesin in vitro by both phosphorylation of threonine 558 and polyphosphatidylinositides. *Mol. Biol. Cell* **10**, 2669–2685 (1999).

18. O. Turunen, T. Wahlstrom, A. Vaheri, Ezrin has a COOH-terminal actin-binding site that is conserved in the ezrin protein family. *J. Cell Biol.* **126**, 1445–1453 (1994).

19. F. Nakamura, M. R. Amieva, C. Hirota, Y. Mizuno, H. Furthmayr, Phosphorylation of 558T of moesin detected by site-specific antibodies in RAW264.7 macrophages. *Biochem. Biophys. Res. Commun.* **226**, 650–656 (1996).

20. K. Hayashi, S. Yonemura, T. Matsui, S. Tsukita, Immunofluorescence detection of ezrin/radixin/moesin (ERM) proteins with their carboxyl-terminal threonine phosphorylated in cultured cells and tissues. *J. Cell Sci.* **112** (Pt. 8), 1149–1158 (1999).

21. S. F. Pietromonaco, P. C. Simons, A. Altman, L. Elias, Protein kinase C-theta phosphorylation of moesin in the actin-binding sequence. *J. Biol. Chem.* **273**, 7594–7603 (1998).

22. A. L. Neisch, R. G. Fehon, Ezrin, Radixin and Moesin: Key regulators of membrane-cortex interactions and signaling. *Curr. Opin. Cell Biol.* **23**, 377–382 (2011).

23. G. T. Charras, C. K. Hu, M. Coughlin, T. J. Mitchison, Reassembly of contractile actin cortex in cell blebs. *J. Cell Biol.* **175**, 477–490 (2006).

24. C. Polesello, F. Payre, Small is beautiful: What flies tell us about ERM protein function in development. *Trends Cell Biol.* **14**, 294–302 (2004).

25. B. M. McCartney, R. G. Fehon, Distinct cellular and subcellular patterns of expression imply distinct functions for the Drosophila homologues of moesin and the neurofibromatosis 2 tumor suppressor, merlin. *J. Cell Biol.* **133**, 843–852 (1996).

26. J. C. Clemens, C. A. Worby, N. Simonson-Leff, M. Muda, T. Maehama, B. A. Hemmings, J. E. Dixon, Use of double-stranded RNA interference in Drosophila cell lines to dissect signal transduction pathways. *Proc. Natl. Acad. Sci. U.S.A.* **97**, 6499–6503 (2000).

27. P. Kunda, A. E. Pelling, T. Liu, B. Baum, Moesin controls cortical rigidity, cell rounding, and spindle morphogenesis during mitosis. *Curr. Biol.* **18**, 91–101 (2008).

28. L. Parisiadou, C. Xie, H. J. Cho, X. Lin, X. L. Gu, C. X. Long, E. Lobbestael, V. Baekelandt, J. M. Taymans, L. Sun, H. Cai, Phosphorylation of ezrin/radixin/moesin proteins by LRRK2 promotes the rearrangement of actin cytoskeleton in neuronal morphogenesis. *J. Neurosci.* **29**, 13971–13980 (2009).

29. D. R. Hipfner, N. Keller, S. M. Cohen, Slik Sterile-20 kinase regulates Moesin activity to promote epithelial integrity during tissue growth. *Genes Dev.* **18**, 2243–2248 (2004).

30. S. C. Hughes, R. G. Fehon, Phosphorylation and activity of the tumor suppressor Merlin and the ERM protein Moesin are coordinately regulated by the Slik kinase. *J. Cell Biol.* **175**, 305–313 (2006).

31. C. Plutoni, S. Keil, C. Zeledon, L. E. A. Delsin, B. Decelle, P. P. Roux, S. Carreno, G. Emery, Misshapen coordinates protrusion restriction and actomyosin contractility during collective cell migration. *Nat. Commun.* **10**, 3940 (2019).

32. C. A. Fu, M. Shen, B. C. Huang, J. Lasaga, D. G. Payan, Y. Luo, TNIK, a novel member of the germinal center kinase family that activates the c-Jun N-terminal kinase pathway and regulates the cytoskeleton. *J. Biol. Chem.* **274**, 30729–30737 (1999).

33. J. E. Treisman, N. Ito, G. M. Rubin, misshapen encodes a protein kinase involved in cell shape control in Drosophila. *Gene* **186**, 119–125 (1997).

34. C. Polesello, I. Delon, P. Valenti, P. Ferrer, F. Payre, Dmoesin controls actin-based cell shape and polarity during Drosophila melanogaster oogenesis. *Nat. Cell Biol.* **4**, 782–789 (2002).

35. B. A. Haubrich, D. C. Swinney, Enzyme activity assays for protein kinases: Strategies to identify active substrates. *Curr. Drug Discov. Technol.* **13**, 2–15 (2016).

36. T. Mahmoudi, V. S. Li, S. S. Ng, N. Taouatas, R. G. Vries, S. Mohammed, A. J. Heck, H. Clevers, The kinase TNIK is an essential activator of Wnt target genes. *EMBO J.* **28**, 3329–3340 (2009).

37. J. Kim, S. H. Moon, B. T. Kim, C. H. Chae, J. Y. Lee, S. H. Kim, A novel aminothiazole KY-05009 with potential to inhibit Traf2- and Nck-interacting kinase (TNIK) attenuates TGF-β1-mediated epithelial-to-mesenchymal transition in human lung adenocarcinoma A549 cells. *PLOS ONE* **9**, e110180 (2014).

38. M. Masuda, Y. Uno, N. Ohbayashi, H. Ohata, A. Mimata, M. Kukimoto-Niino, H. Moriyama, S. Kashimoto, T. Inoue, N. Goto, K. Okamoto, M. Shirouzu, M. Sawa, T. Yamada, TNIK inhibition abrogates colorectal cancer stemness. *Nat. Commun.* **7**, 12586 (2016).

39. Q. Wang, S. P. Amato, D. M. Rubitski, M. M. Hayward, B. L. Kormos, P. R. Verhoest, L. Xu, N. J. Brandon, M. D. Ehlers, Identification of phosphorylation consensus sequences and endogenous neuronal substrates of the psychiatric risk kinase TNIK. *J. Pharmacol. Exp. Ther.* **356**, 410–423 (2016).

40. A. A. Zarban, H. Chaudhry, D. Maselli, X. Kodji, J. de Sousa Valente, J. Joachim, S. C. Trevelin, J. van Baardewijk, F. Argunhan, A. Ivetic, M. Nandi, S. D. Brain, Enhancing techniques for determining inflammatory edema formation and neutrophil accumulation in murine skin. *JID Innov.* **3**, 100154 (2023).

41. J. T. Brash, C. Ruhrberg, A. Fantin, Evaluating vascular hyperpermeability-inducing agents in the skin with the Miles assay. *J. Vis. Exp.*, 10.3791/57524 (2018).

42. R. H. Poyser, G. B. West, Changes in vascular permeability produced in rats by dextran, ovomucoid and yeast cell wall polysaccharides. *Br. J. Pharmacol. Chemother.* **25**, 602–609 (1965).

43. M. Finsterbusch, M. B. Voisin, M. Beyrau, T. J. Williams, S. Nourshargh, Neutrophils recruited by chemoattractants in vivo induce microvascular plasma protein leakage through secretion of TNF. *J. Exp. Med.* **211**, 1307–1314 (2014).

44. M. Brandt, V. Gerke, T. Betz, Human endothelial cells display a rapid tensional stress increase in response to tumor necrosis factor-α. *PLOS ONE* **17**, e0270197 (2022).

45. T. Kihara, T. Matsumoto, Y. Nakahashi, K. Tachibana, Mechanical stiffness softening and cell adhesion are coordinately regulated by ERM dephosphorylation in KG-1 cells. *Hum. Cell* **34**, 1709–1716 (2021).

46. Y. Liu, N. V. Belkina, C. Park, R. Nambiar, S. M. Loughhead, G. Patino-Lopez, K. Ben-Aissa, J. J. Hao, M. J. Kruhlak, H. Qi, U. H. von Andrian, J. H. Kehrl, M. J. Tyska, S. Shaw, Constitutively active ezrin increases membrane tension, slows migration, and impedes endothelial transmigration of lymphocytes in vivo in mice. *Blood* **119**, 445–453 (2012).

47. B. Rouven Bruckner, A. Pietuch, S. Nehls, J. Rother, A. Janshoff, Ezrin is a major regulator of membrane tension in epithelial cells. *Sci. Rep.* **5**, 14700 (2015).

48. G. Fu, Q. Xu, Y. Qiu, X. Jin, T. Xu, S. Dong, J. Wang, Y. Ke, H. Hu, X. Cao, D. Wang, H. Cantor, X. Gao, L. Lu, Suppression of Th17 cell differentiation by misshapen/NIK-related kinase MINK1. *J. Exp. Med.* **214**, 1453–1469 (2017).

49. B. Nicke, J. Bastien, S. J. Khanna, P. H. Warne, V. Cowling, S. J. Cook, G. Peters, O. Delpuech, A. Schulze, K. Berns, J. Mullenders, R. L. Beijersbergen, R. Bernards, T. S. Ganesan, J. Downward, D. C. Hancock, Involvement of MINK, a Ste20 family kinase, in Ras oncogene-induced growth arrest in human ovarian surface epithelial cells. *Mol. Cell* **20**, 673–685 (2005).

50. L. R. Fiedler, K. Chapman, M. Xie, E. Maifoshie, M. Jenkins, P. A. Golforoush, M. Bellahcene, M. Noseda, D. Faust, A. Jarvis, G. Newton, M. A. Paiva, M. Harada, D. J. Stuckey, W. Song, J. Habib, P. Narasimham, R. Aqil, D. Sanmugalingam, R. Yan, L. Pavanello, M. Sano, S. C. Wang, R. D. Sampson, S. Kanayaganam, G. E. Taffet, L. H. Michael, M. L. Entman, T. H. Tan, S. E. Harding, C. M. R. Low, C. Tralau-Stewart, T. Perrior, M. D. Schneider, MAP4K4 inhibition promotes survival of human stem cell-derived cardiomyocytes and reduces infarct size in vivo. *Cell Stem Cell* **24**, 579–591.e12 (2019).

51. J. R. Burgoyne, O. Oviosu, P. Eaton, The PEG-switch assay: A fast semi-quantitative method to determine protein reversible cysteine oxidation. *J. Pharmacol. Toxicol. Methods* **68**, 297–301 (2013).

52. F. Antunes, P. M. Brito, Quantitative biology of hydrogen peroxide signaling. *Redox Biol.* **13**, 1–7 (2017).

53. A. Corcoran, T. G. Cotter, Redox regulation of protein kinases. *FEBS J.* **280**, 1944–1965 (2013).

54. R. Sanchez, M. Riddle, J. Woo, J. Momand, Prediction of reversibly oxidized protein cysteine thiols using protein structure properties. *Protein Sci.* **17**, 473–481 (2008).

55. I. Soylu, S. M. Marino, Cy-preds: An algorithm and a web service for the analysis and prediction of cysteine reactivity. *Proteins* **84**, 278–291 (2016).

56. E. Krissinel, K. Henrick, Inference of macromolecular assemblies from crystalline state. *J. Mol. Biol.* **372**, 774–797 (2007).

57. A. Mikryukov, T. Moss, Agonistic and antagonistic roles for TNIK and MINK in non-canonical and canonical Wnt signalling. *PLOS ONE* **7**, e43330 (2012).

58. B. Aquino, R. M. Counago, N. Verza, L. M. Ferreira, K. B. Massirer, O. Gileadi, P. Arruda, Structural characterization of Maize SIRK1 kinase domain reveals an unusual architecture of the activation segment. *Front. Plant Sci.* **8**, 852 (2017).

59. K. Tsujita, R. Satow, S. Asada, Y. Nakamura, L. Arnes, K. Sako, Y. Fujita, K. Fukami, T. Itoh, Homeostatic membrane tension constrains cancer cell dissemination by counteracting BAR protein assembly. *Nat. Commun.* **12**, 5930 (2021).

60. K. Tachibana, S. M. Haghparast, J. Miyake, Inhibition of cell adhesion by phosphorylated Ezrin/Radixin/Moesin. *Cell Adh. Migr.* **9**, 502–512 (2015).

61. F. Freitas, E. Tibirica, M. Singh, P. A. Fraser, G. E. Mann, Redox regulation of microvascular permeability: IL-1beta potentiation of bradykinin-induced permeability is prevented by simvastatin. *Antioxidants* **9**,1269 (2020).

62. E. Panieri, M. M. Santoro, ROS signaling and redox biology in endothelial cells. *Cell. Mol. Life Sci.* **72**, 3281–3303 (2015).

63. R. S. Frey, A. Rahman, J. C. Kefer, R. D. Minshall, A. B. Malik, PKCzeta regulates TNF-alpha-induced activation of NADPH oxidase in endothelial cells. *Circ. Res.* **90**, 1012–1019 (2002).

64. C. Cui, M. Jiang, N. Jain, S. Das, Y. H. Lo, A. A. Kermani, T. Pipatpolkai, J. Sun, Structural basis of human NOX5 activation. *Nat. Commun.* **15**, 3994 (2024).

65. Y. Nisimoto, B. A. Diebold, D. Cosentino-Gomes, J. D. Lambeth, Nox4: A hydrogen peroxide-generating oxygen sensor. *Biochemistry* **53**, 5111–5120 (2014).

66. L. Serrander, L. Cartier, K. Bedard, B. Banfi, B. Lardy, O. Plastre, A. Sienkiewicz, L. Forro, W. Schlegel, K. H. Krause, NOX4 activity is determined by mRNA levels and reveals a unique pattern of ROS generation. *Biochem. J.* **406**, 105–114 (2007).

67. D. X. Zhang, D. D. Gutterman, Mitochondrial reactive oxygen species-mediated signaling in endothelial cells. *Am. J. Physiol. Heart Circ. Physiol.* **292**, H2023–H2031 (2007).

68. H. He, J. J. Mack, E. Guc, C. M. Warren, M. L. Squadrito, W. W. Kilarski, C. Baer, R. D. Freshman, A. I. McDonald, S. Ziyad, M. A. Swartz, M. De Palma, M. L. Iruela-Arispe, Perivascular macrophages limit permeability. *Arterioscler. Thromb. Vasc. Biol.* **36**, 2203–2212 (2016).

69. A. Lapenna, M. De Palma, C. E. Lewis, Perivascular macrophages in health and disease. *Nat. Rev. Immunol.* **18**, 689–702 (2018).

70. K. Taira, M. Umikawa, K. Takei, B. E. Myagmar, M. Shinzato, N. Machida, H. Uezato, S. Nonaka, K. Kariya, The Traf2- and Nck-interacting kinase as a putative effector of Rap2 to regulate actin cytoskeleton. *J. Biol. Chem.* **279**, 49488–49496 (2004).

71. Y. Uechi, M. Bayarjargal, M. Umikawa, M. Oshiro, K. Takei, Y. Yamashiro, T. Asato, S. Endo, R. Misaki, T. Taguchi, K. Kariya, Rap2 function requires palmitoylation and recycling endosome localization. *Biochem. Biophys. Res. Commun.* **378**, 732–737 (2009).

72. H. Nonaka, K. Takei, M. Umikawa, M. Oshiro, K. Kuninaka, M. Bayarjargal, T. Asato, Y. Yamashiro, Y. Uechi, S. Endo, T. Suzuki, K. I. Kariya, MINK is a Rap2 effector for phosphorylation of the postsynaptic scaffold protein TANC1. *Biochem. Biophys. Res. Commun.* **377**, 573–578 (2008).

73. N. Machida, M. Umikawa, K. Takei, N. Sakima, B. E. Myagmar, K. Taira, H. Uezato, Y. Ogawa, K. Kariya, Mitogen-activated protein kinase kinase kinase kinase 4 as a putative effector of Rap2 to activate the c-Jun N-terminal kinase. *J. Biol. Chem.* **279**, 15711–15714 (2004).

74. M. Gloerich, J. P. ten Klooster, M. J. Vliem, T. Koorman, F. J. Zwartkruis, H. Clevers, J. L. Bos, Rap2A links intestinal cell polarity to brush border formation. *Nat. Cell Biol.* **14**, 793–801 (2012).

75. W. J. Pannekoek, J. R. Linnemann, P. M. Brouwer, J. L. Bos, H. Rehmann, Rap1 and Rap2 antagonistically control endothelial barrier resistance. *PLOS ONE* **8**, e57903 (2013).

76. R. J. Roth Flach, A. Skoura, A. Matevossian, L. V. Danai, W. Zheng, C. Cortes, S. K. Bhattacharya, M. Aouadi, N. Hagan, J. C. Yawe, P. Vangala, L. G. Menendez, M. P. Cooper, T. P. Fitzgibbons, L. Buckbinder, M. P. Czech, Endothelial protein kinase MAP4K4 promotes vascular inflammation and atherosclerosis. *Nat. Commun.* **6**, 8995 (2015).

77. L. Botros, M. C. A. P. Pronk, J. M. Juschten, J. Liddle, S. Morsing, J. D. P. van Buul, R. H. Bates, P. R. M. D. P. Tuinman, J. S. M. van Bezu, S. P. Huveneers, H. J. M. D. P. Bogaard, V. W. M. P. van Hinsbergh, P. L. P. Hordijk, J. M. D. P. Aman, Bosutinib prevents vascular leakage by reducing focal adhesion turnover and reinforcing junctional integrity. *J. Cell Sci.* **133**, jcs240077 (2020).

78. M. Baumgartner, A. L. Sillman, E. M. Blackwood, J. Srivastava, N. Madson, J. W. Schilling, J. H. Wright, D. L. Barber, The Nck-interacting kinase phosphorylates ERM proteins for formation of lamellipodium by growth factors. *Proc. Natl. Acad. Sci. U.S.A.* **103**, 13391–13396 (2006).

79. Y. Wang, M. Nakayama, M. E. Pitulescu, T. S. Schmidt, M. L. Bochenek, A. Sakakibara, S. Adams, A. Davy, U. Deutsch, U. Luthi, A. Barberis, L. E. Benjamin, T. Makinen, C. D. Nobes, R. H. Adams, Ephrin-B2 controls VEGF-induced angiogenesis and lymphangiogenesis. *Nature* **465**, 483–486 (2010).

80. S. Brown, Institutional profile: The Sheffield RNAi screening facility: A service for high-throughput, genome-wide *Drosophila* RNAi screens. *Future Med. Chem.* **2**, 1805–1812 (2010).

81. T. Horn, T. Sandmann, M. Boutros, Design and evaluation of genome-wide libraries for RNA interference screens. *Genome Biol.* **11**, R61 (2010).

82. J. K. Eng, A. L. McCormack, J. R. Yates, An approach to correlate tandem mass spectral data of peptides with amino acid sequences in a protein database. *J. Am. Soc. Mass Spectrom.* **5**, 976–989 (1994).

83. M. Biasini, S. Bienert, A. Waterhouse, K. Arnold, G. Studer, T. Schmidt, F. Kiefer, T. Gallo Cassarino, M. Bertoni, L. Bordoli, T. Schwede, SWISS-MODEL: Modelling protein tertiary and quaternary structure using evolutionary information. *Nucleic Acids Res.* **42**, W252–W258 (2014).

84. D. Kozakov, D. R. Hall, B. Xia, K. A. Porter, D. Padhorny, C. Yueh, D. Beglov, S. Vajda, The ClusPro web server for protein-protein docking. *Nat. Protoc.* **12**, 255–278 (2017).

85. D. E. Pires, D. B. Ascher, T. L. Blundell, DUET: A server for predicting effects of mutations on protein stability using an integrated computational approach. *Nucleic Acids Res.* **42**, W314–W319 (2014).

86. J. A. Maier, C. Martinez, K. Kasavajhala, L. Wickstrom, K. E. Hauser, C. Simmerling, ff14SB: Improving the accuracy of protein side chain and backbone parameters from ff99SB. *J. Chem. Theory Comput.* **11**, 3696–3713 (2015).

87. M. Mahoney, W. Jorgensen, A five-site model for liquid water and the reproduction of the density anomaly by rigid. *J. Chem. Phys.* **112**, 8910–8922 (2000).

88. G. Bussi, D. Donadio, M. Parrinello, Canonical sampling through velocity rescaling. *J. Chem. Phys.* **126**, 014101 (2007).

89. H. J. C. Berendsen, J. P. M. Postma, W. F. van Gunsteren, A. DiNola, J. R. Haak, Molecular dynamics with coupling to an external bath. *J. Chem. Phys.* **81**, 3684–3690 (1984).

90. M. Parrinello, A. Rahman, Polymorphic transitions in single crystals: A new molecular dynamics method. *J. Appl. Phys.* **52**, 7182–7190 (1981).

91. T. Darden, D. York, L. Pedersen, Particle mesh Ewald: An $N \cdot \log(N)$ method for Ewald sums in large systems. *J. Chem. Phys.* **98**, 10089–10092 (1993).

92. B. Hess, H. Bekker, H. J. C. Berendsen, J. G. E. M. Fraaije, LINCS: A linear constraint solver for molecular simulations. *J. Comput. Chem.* **18**, 1463–1472 (1997).

93. W. Humphrey, A. Dalke, K. Schulten, VMD: Visual molecular dynamics. *J. Mol. Graph.* **14**, 33–38 (1996).

94. J. Kleinjung, F. Fraternali, POPSCOMP: An automated interaction analysis of biomolecular complexes. *Nucleic Acids Res.* **33**, W342–W346 (2005).

95. P. Swiatlowska, J. L. Sanchez-Alonso, C. Mansfield, D. Scaini, Y. Korchev, P. Novak, J. Gorelik, Short-term angiotensin II treatment regulates cardiac nanomechanics via microtubule modifications. *Nanoscale* **12**, 16315–16329 (2020).

96. I. Sawyer, S. J. Smillie, J. V. Bodkin, E. Fernandes, K. T. O'Byrne, S. D. Brain, The vasoactive potential of kisspeptin-10 in the peripheral vasculature. *PLOS ONE* **6**, e14671 (2011).

97. P. P. Bradley, D. A. Priebat, R. D. Christensen, G. Rothstein, Measurement of cutaneous inflammation: Estimation of neutrophil content with an enzyme marker. *J. Invest. Dermatol.* **78**, 206–209 (1982).

98. A. Rey-Gallardo, H. Tomlins, J. Joachim, I. Rahman, P. Kitscha, K. Frudd, M. Parsons, A. Ivetic, Sequential binding of ezrin and moesin to L-selectin regulates monocyte protrusive behaviour during transendothelial migration. *J. Cell Sci.* **131**, (2018).

99. D. J. Chambers, H. B. Fallouh, Cardioplegia and cardiac surgery: Pharmacological arrest and cardioprotection during global ischemia and reperfusion. *Pharmacol. Ther.* **127**, 41–52 (2010).

100. A. W. Oliver, S. Knapp, L. H. Pearl, Activation segment exchange: A common mechanism of kinase autophosphorylation? *Trends Biochem. Sci.* **32**, 351–356 (2007).

101. D. Marcotte, M. Rushe, R. M. Arduini, C. Lukacs, K. Atkins, X. Sun, K. Little, M. Cullivan, M. Paramasivam, T. A. Patterson, T. Hesson, T. D. Mc Kee, T. L. May-Dracka, Z. Xin, A. Bertolotti-Ciarlet, G. R. Bhisetti, J. P. Lyssikatos, L. F. Silvian, Germinal-center kinase-like

kinase co-crystal structure reveals a swapped activation loop and C-terminal extension. *Protein Sci.* **26**, 152–162 (2017).

102. L. Wang, M. Stanley, J. W. Boggs, T. D. Crawford, B. J. Bravo, A. M. Giannetti, S. F. Harris, S. R. Magnuson, J. Nonomiya, S. Schmidt, P. Wu, W. Ye, S. E. Gould, L. J. Murray, C. O. Ndubaku, H. Chen, Fragment-based identification and optimization of a class of potent pyrrolo[2,1-f][1,2,4]triazine MAP4K4 inhibitors. *Bioorg. Med. Chem. Lett.* **24**, 4546–4552 (2014).

103. A. C. Pike, P. Rellos, F. H. Niesen, A. Turnbull, A. W. Oliver, S. A. Parker, B. E. Turk, L. H. Pearl, S. Knapp, Activation segment dimerization: A mechanism for kinase autophosphorylation of non-consensus sites. *EMBO J.* **27**, 704–714 (2008).

104. C. J. Record, A. Chaikuad, P. Rellos, S. Das, A. C. Pike, O. Fedorov, B. D. Marsden, S. Knapp, W. H. Lee, Structural comparison of human mammalian ste20-like kinases. *PLOS ONE* **5**, e11905 (2010).

105. N. V. Belkina, Y. Liu, J. J. Hao, H. Karasuyama, S. Shaw, LOK is a major ERM kinase in resting lymphocytes and regulates cytoskeletal rearrangement through ERM phosphorylation. *Proc. Natl. Acad. Sci. U.S.A.* **106**, 4707–4712 (2009).

106. M. Machicoane, C. A. de Frutos, J. Fink, M. Rocancourt, Y. Lombardi, S. Garel, M. Piel, A. Echard, SLK-dependent activation of ERMs controls LGN-NuMA localization and spindle orientation. *J. Cell Biol.* **205**, 791–799 (2014).

107. J. P. ten Klooster, M. Jansen, J. Yuan, V. Oorschot, H. Begthel, V. Di Giacomo, F. Colland, J. de Koning, M. M. Maurice, P. Hornbeck, H. Clevers, Mst4 and Ezrin induce brush borders downstream of the Lkb1/Strad/Mo25 polarization complex. *Dev. Cell* **16**, 551–562 (2009).

108. A. Lupas, M. Van Dyke, J. Stock, Predicting coiled coils from protein sequences. *Science* **252**, 1162–1164 (1991).

109. A. Y. Luhovy, A. Jaberi, J. Papillon, J. Guillemette, A. V. Cybulsky, Regulation of the Ste20-like kinase, SLK: Involvement of activation segment phosphorylation. *J. Biol. Chem.* **287**, 5446–5458 (2012).
